# Supplementary material for: Single molecule magnet with an unpaired electron trapped between two lanthanide ions inside a fullerene
Source: Nat Commun. 2017 Jul 14;8:16098. doi: 10.1038/ncomms16098 (PMC5519982; doi:10.1038/ncomms16098)
Supplement: Supplementary Information [file ncomms16098-s1.pdf]

Title of file for HTML: Supplementary Information
Description: Supplementary Figures, Supplementary Tables, Supplementary Notes and Supplementary References

Title of file for HTML: Peer Review File
Description:

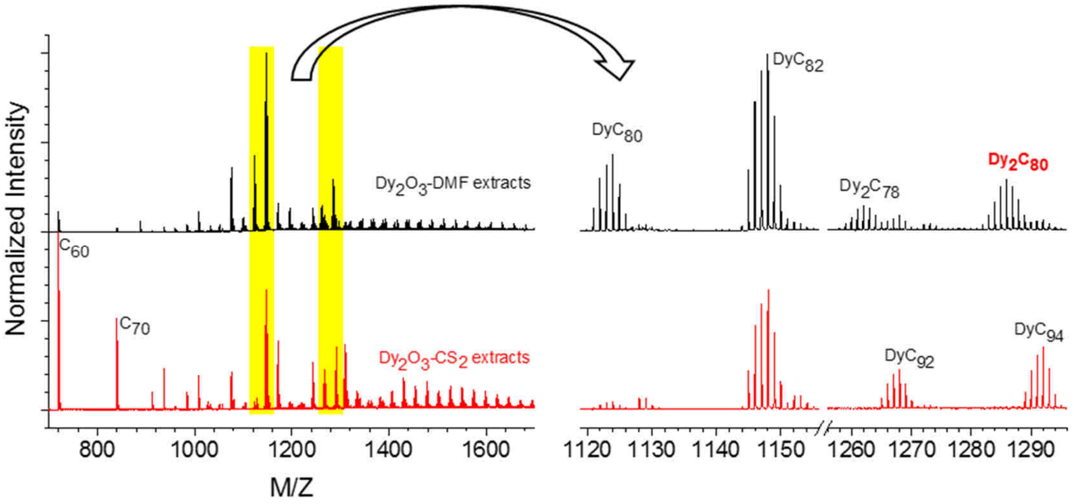

Supplementary Figure 1. Comparison of the mass-spectra of Dy-EMF extracts obtained with DMF and CS_2 solvents. DMF extracts both monometallofullerenes and dimetallofullerenes, whereas the CS_2 extracts only monometallofullerenes.

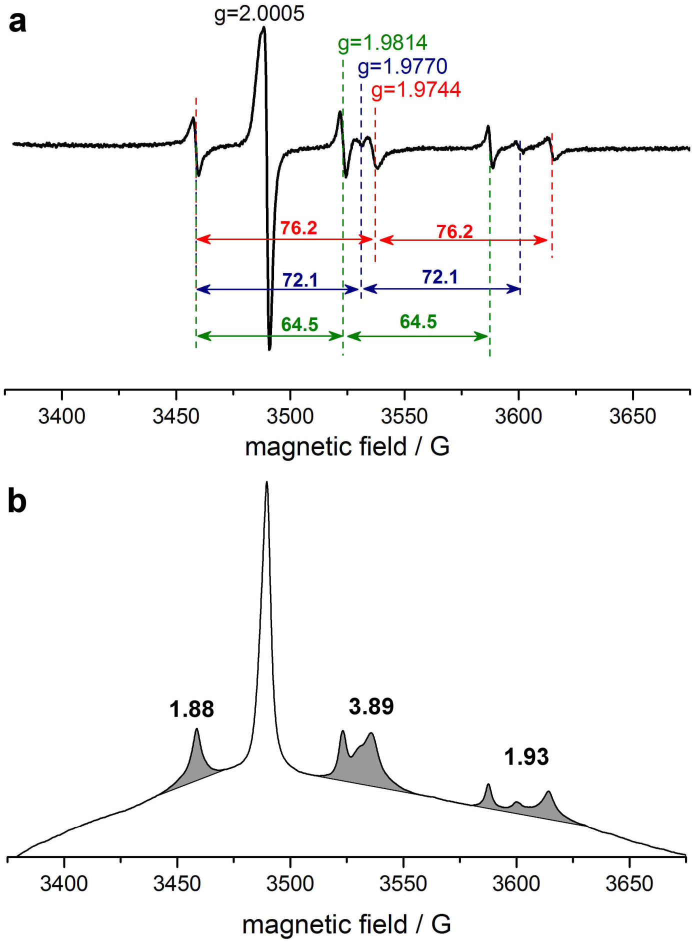

Supplementary Figure 2. Electron paramagnetic resonance spectra of Y-EMF extract in DMF. (a) the measured spectrum (derivative of the absorption); (b) an integral of the spectrum in (a). Shaded areas and numbers show double integrals (proportional to the number of spins) for the peaks assigned to $Y_2@C_{2n}^-$ anion radicals.

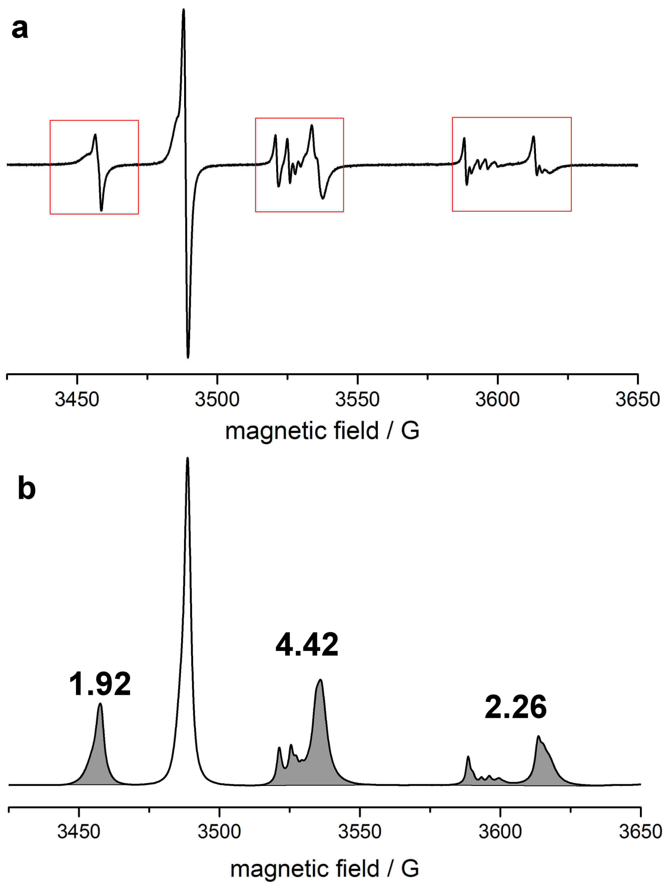

Supplementary Figure 3. Electron paramagnetic resonance spectrum of the mixture of benzyl adducts of Y-EMFs dissolved in toluene. (a) The spectrum of the derivatized $Y_2@C_{2n}$ is more complex than that of the DMF extract, showing that several isomers of monoadducts are formed; (b) integral of the spectrum in (a), showing the ratio of integrated areas. It is still close to 1:2:1, showing that the hyperfine structure with large hfc constants is due to the presence of two Y nuclei in each radical with large but probably not equal $a(^{89}Y)$ values.

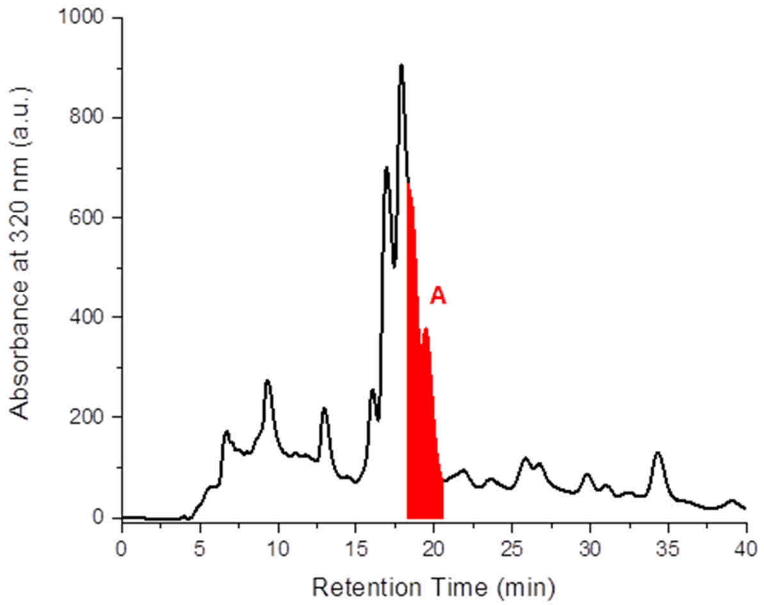

Supplementary Figure 4. Chromatogram of the mixture of benzyl-derivatized Dy-EMFs. Fraction marked A contained $Dy_2@C_{80}(CH_2Ph)$ derivatives (main components however are $Dy@C_{82}(CH_2Ph)$ derivatives, ca 10 isomer thereof are formed). HPLC conditions: linear combination of two 4.6 × 250 mm Buckyprep columns; flow rate 1.6 mL/min; injection volume 800 μL; toluene as eluent; 40 °C

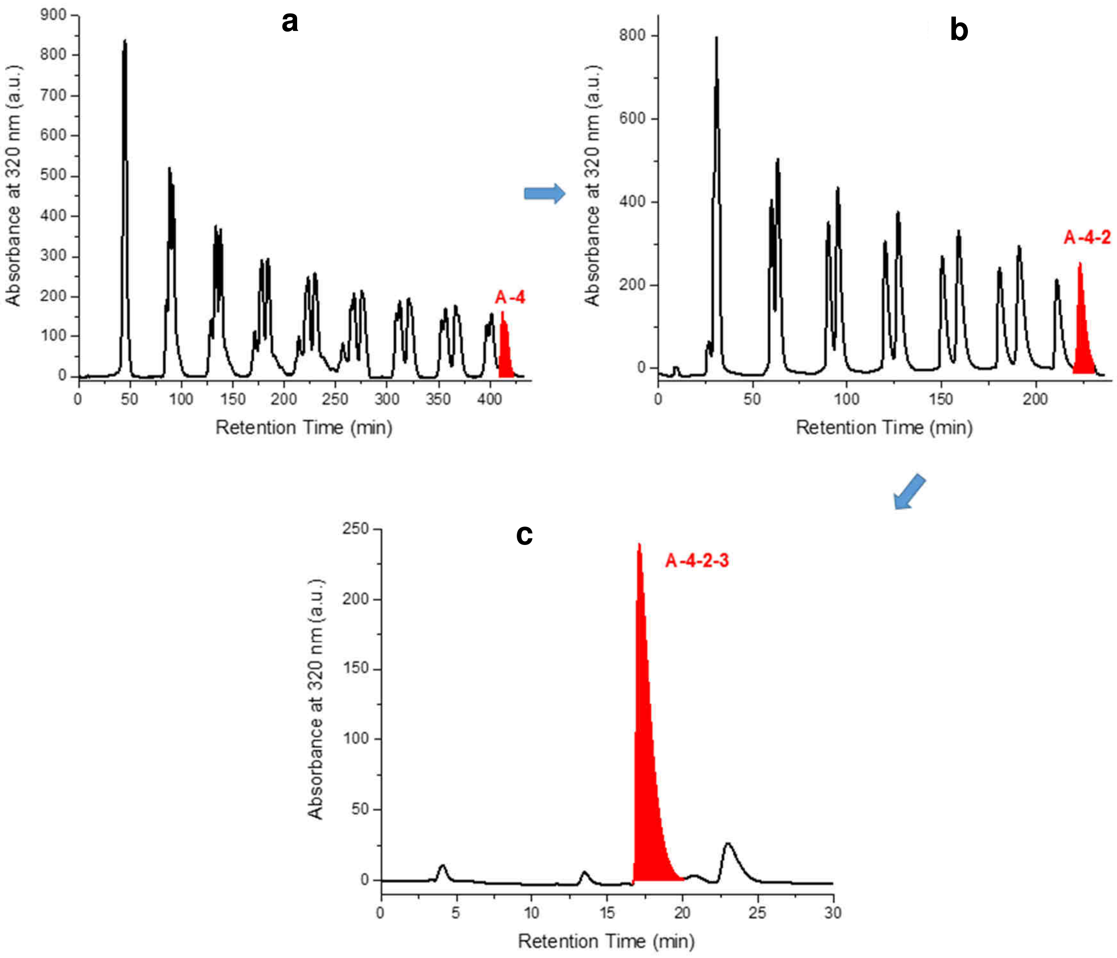

Supplementary Figure 5. Separation of **Dy_2-I**. Three steps were required to obtain pure **Dy_2-I** from fraction A. **(a)** Recycling HPLC profile of fraction A (10 × 250 mm Buckyprep column; flow rate 2 mL/min; injection volume 4.5 mL; toluene as eluent). **(b)** Recylcing HPLC profile of fraction A-4 (10 × 250 mm Buckyprep-M column; flow rate 2 mL/min; injection volume 4.5 mL; toluene as eluent). **(c)** HPLC profile of fraction A-4-2 (4.6 × 250 mm Buckyprep-D column; flow rate 1.0 mL/min; injection volume 500 μL; toluene as eluent; 40 °C). Pure Dy_2-I was obtained as ϕ fraction A-4-2-3.

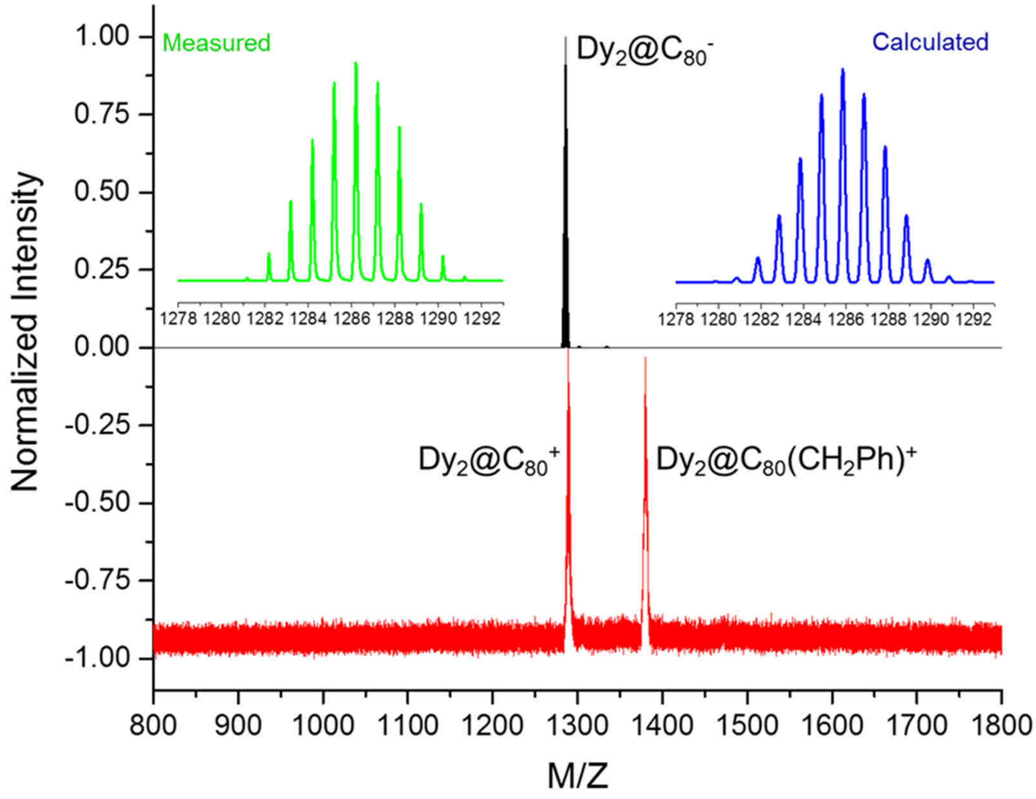

Supplementary Figure 6. Matrix-assisted laser desorption-ionization time-of-flight mass-spectra of **Dy_2-I**. Linear negative (top) and positive (bottom) ionization modes, 1,1,4,4-tetraphenyl-1,3-butadiene was used as matrix. Resolution in positive mode is not high enough for analysis of isotopic distribution. In the negative ion mode, strong fragmentation does not allow for detection of molecular peak, but spectral resolution is sufficient to prove correct isotopic distribution of the $Dy_2@C_{80}^-$ fragment.

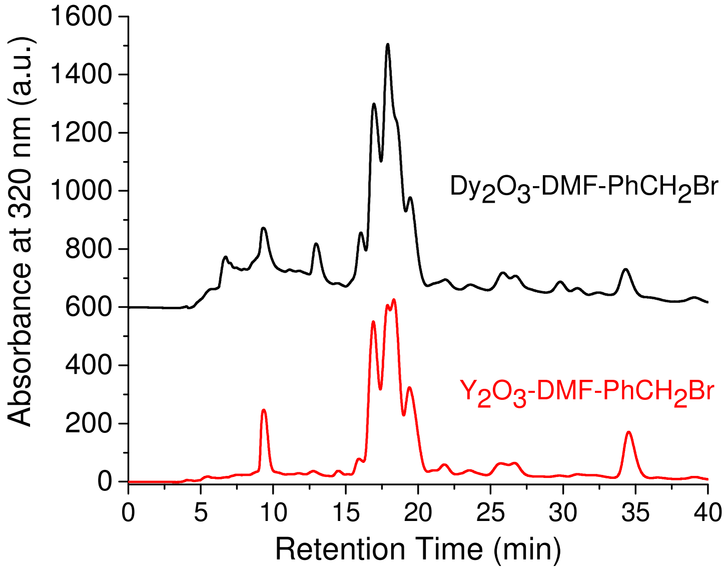

Supplementary Figure 7. Comparison of the chromatograms of Dy-EMFs and Y-EMF DMF extracts after benzyl-derivatization. HPLC conditions: linear combination of two 4.6 × 250 mm Buckyprep columns; flow rate 1.6 mL/min; injection volume 800 μL; toluene as eluent; 40 °C

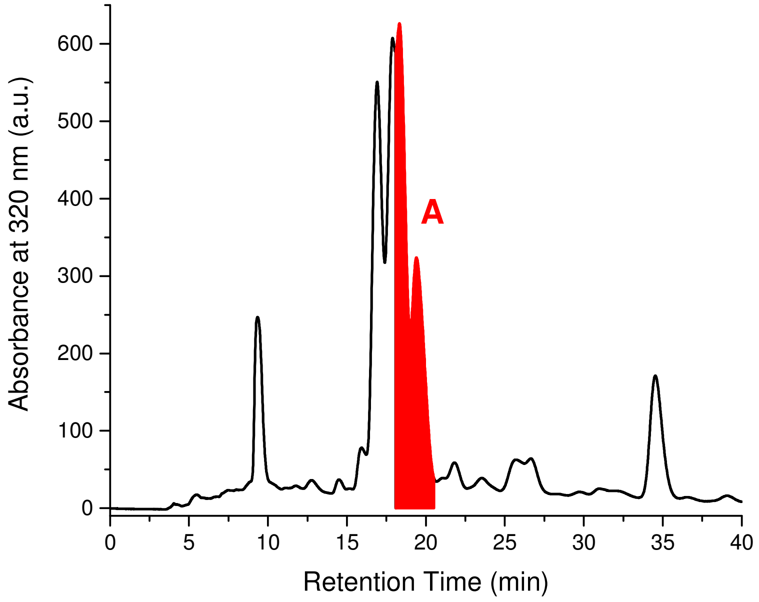

Supplementary Figure 8. Chromatogram of the mixture of benzyl-derivatized Y-EMFs. Fraction A contained $Y_2@C_{80}(CH_2Ph)$ derivative (main components however are $Y@C_{82}(CH_2Ph)$ derivatives, ca 10 isomer thereof are formed). HPLC conditions: linear combination of two 4.6 × 250 mm Buckyprep columns; flow rate 1.6 mL/min; injection volume 800 μL; toluene as eluent; 40 °C

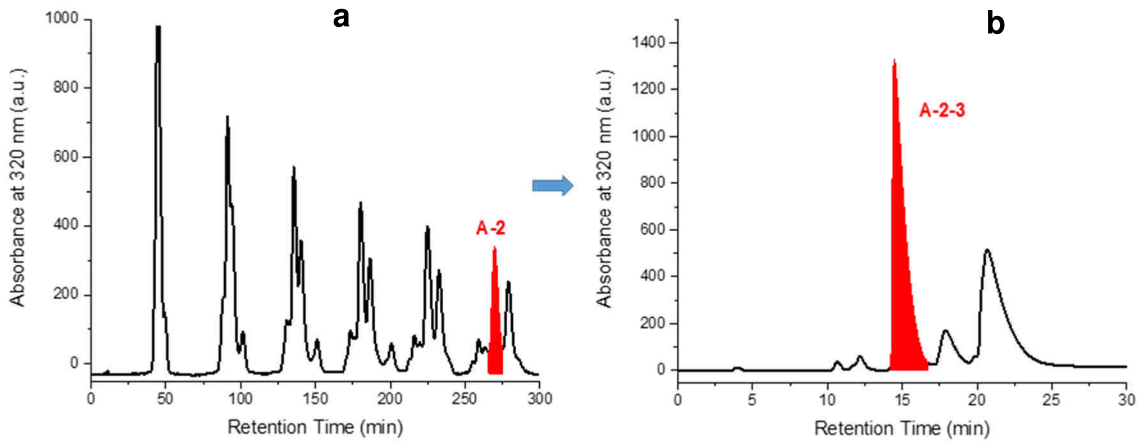

Supplementary Figure 9. Separation of $\mathbf{Y_2}$**-I**. Two steps were required to obtain pure $\mathbf{Y_2}$**-I**. **(a)** Recycling HPLC profile of fraction A (10 × 250 mm Buckyprep column; flow rate 2 mL/min; injection volume 4.5 mL; toluene as eluent). **(b)** HPLC profile of fraction A-2 (4.6 × 250 mm Buckyprep-D column; flow rate 1.0 mL/min; injection volume 500 μL; toluene as eluent; 40 °C). Pure Y_2-I was obtained as fraction A-2-3.

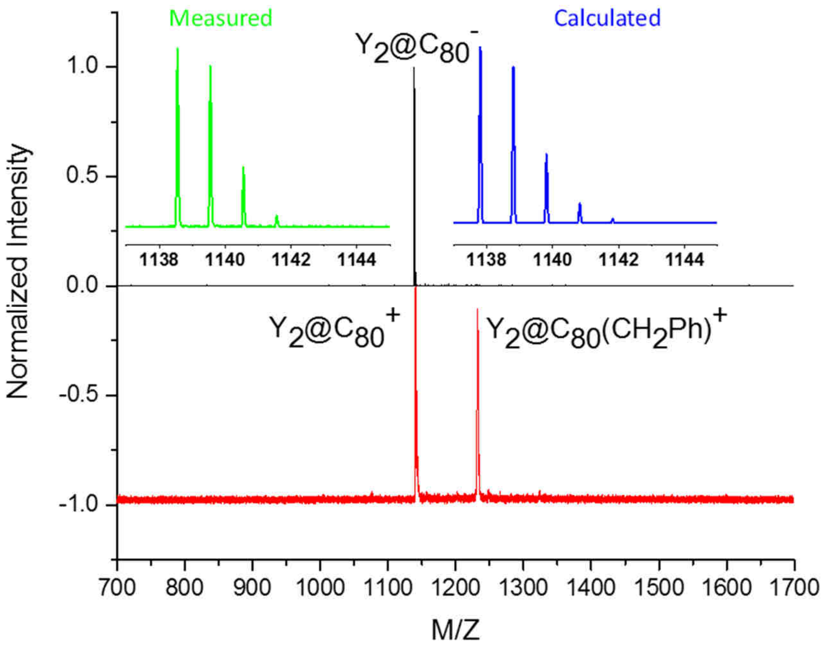

Supplementary Figure 10. . Matrix-assisted laser desorption-ionization time-of-flight mass-spectra of **Y_2-I**. Linear negative (top) and positive (bottom) ionization modes, 1,1,4,4-tetraphenyl-1,3-butadiene was used as matrix. Resolution in positive mode is not high enough for analysis of isotopic distribution. In the negative ion mode, strong fragmentation does not allow for detection of molecular peak, but spectral resolution is sufficient to prove correct isotopic distribution of the $Y_2@C_{80}^-$ fragment.

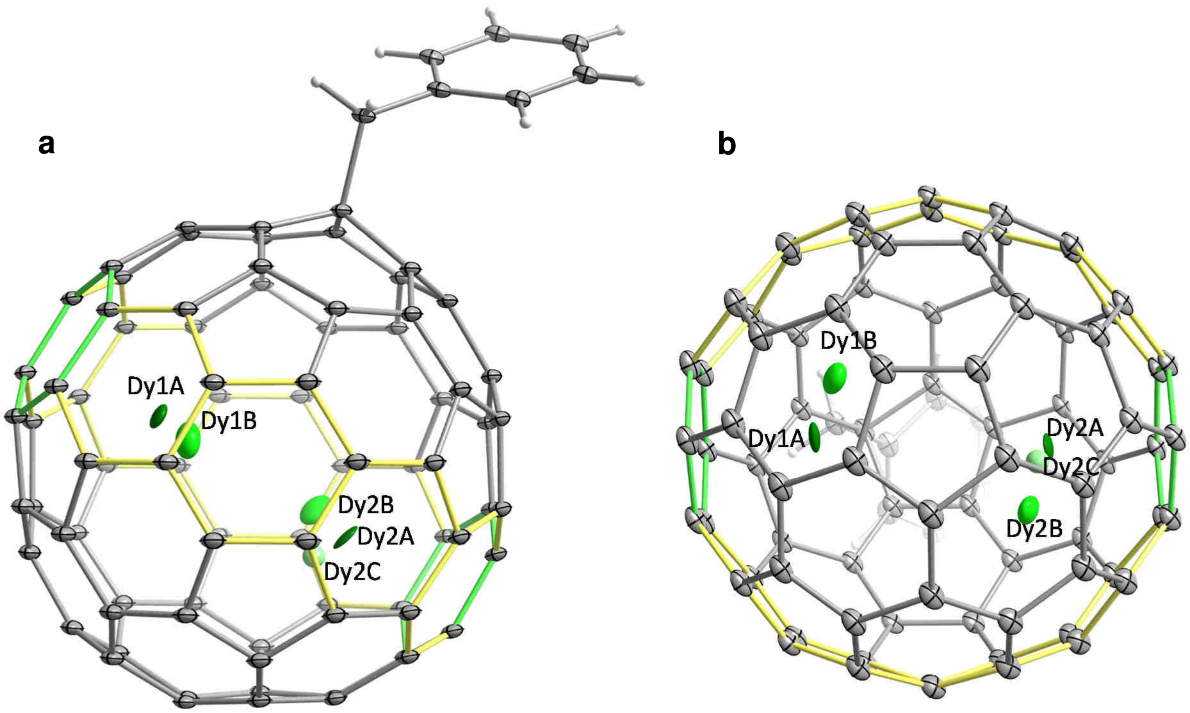

Supplementary Figure 11. Molecular structure of **Dy_2-I** from single-crystal X-ray diffraction. **(a)** Molecular structure shown with 50% thermal ellipsoids. Two hexagons coordinating the main metal atoms are highlighted green. **(b)** Same as (a) but showing the molecule in a different orientation.

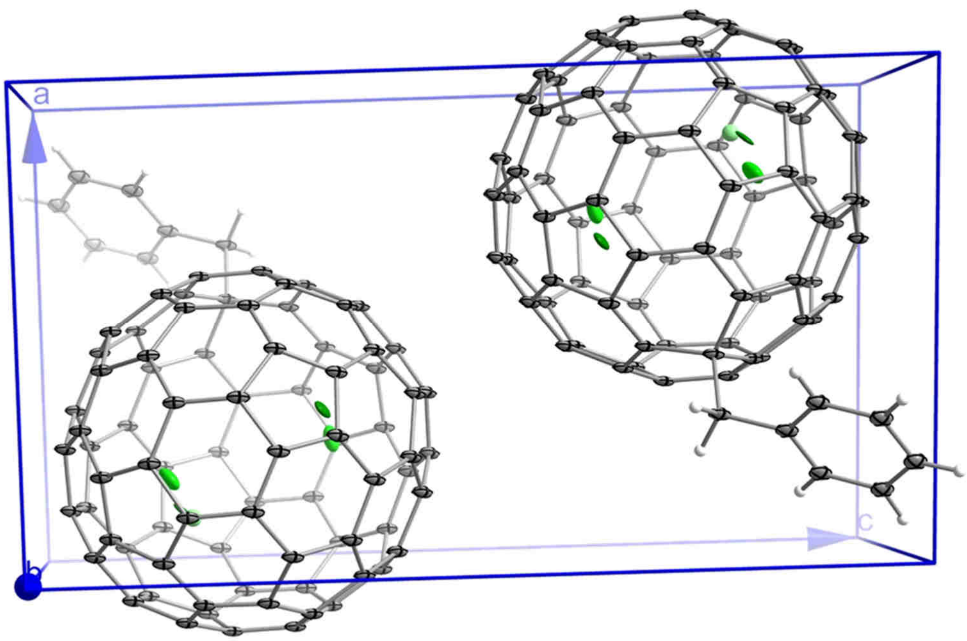

Supplementary Figure 12. Packing of the **Dy_2-I** molecules in the unit cell.

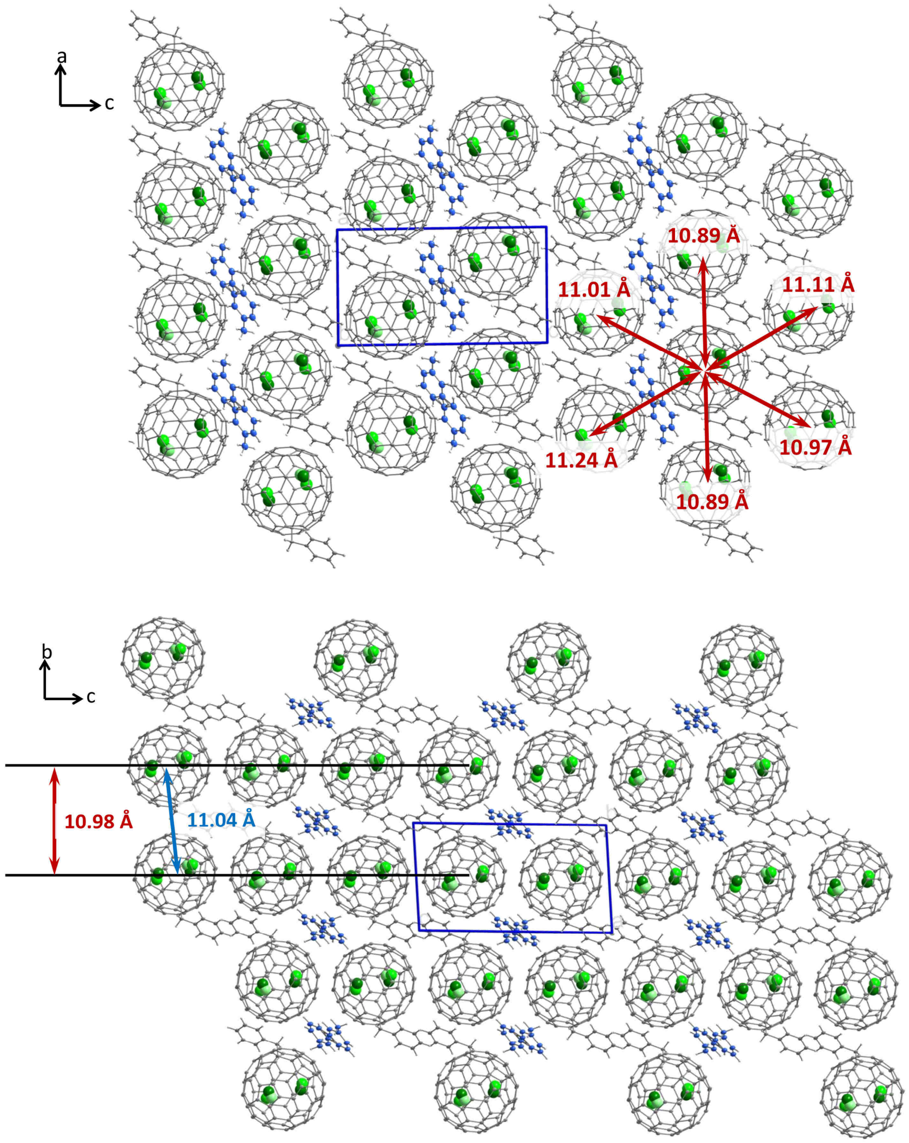

Supplementary Figure 13. Packing of the **Dy_2-I** molecules in the crystal. Top: View along the *b* axis. Bottom - view along the *a* axis. All atoms are shown with 50% thermal ellipsoids except for Dy atoms, which are shown with ball model. The main position of Dy atoms with larger occupancies are shown with green ball, Dy atoms with lower occupancies are shown as light green ball, and the Dy atoms with the lowest occupancies were shown as pale green ball. The carbon atoms of toluene molecules in the voids are highlighted with light blue. Also shows are the distances between centroids of fullerene molecules.

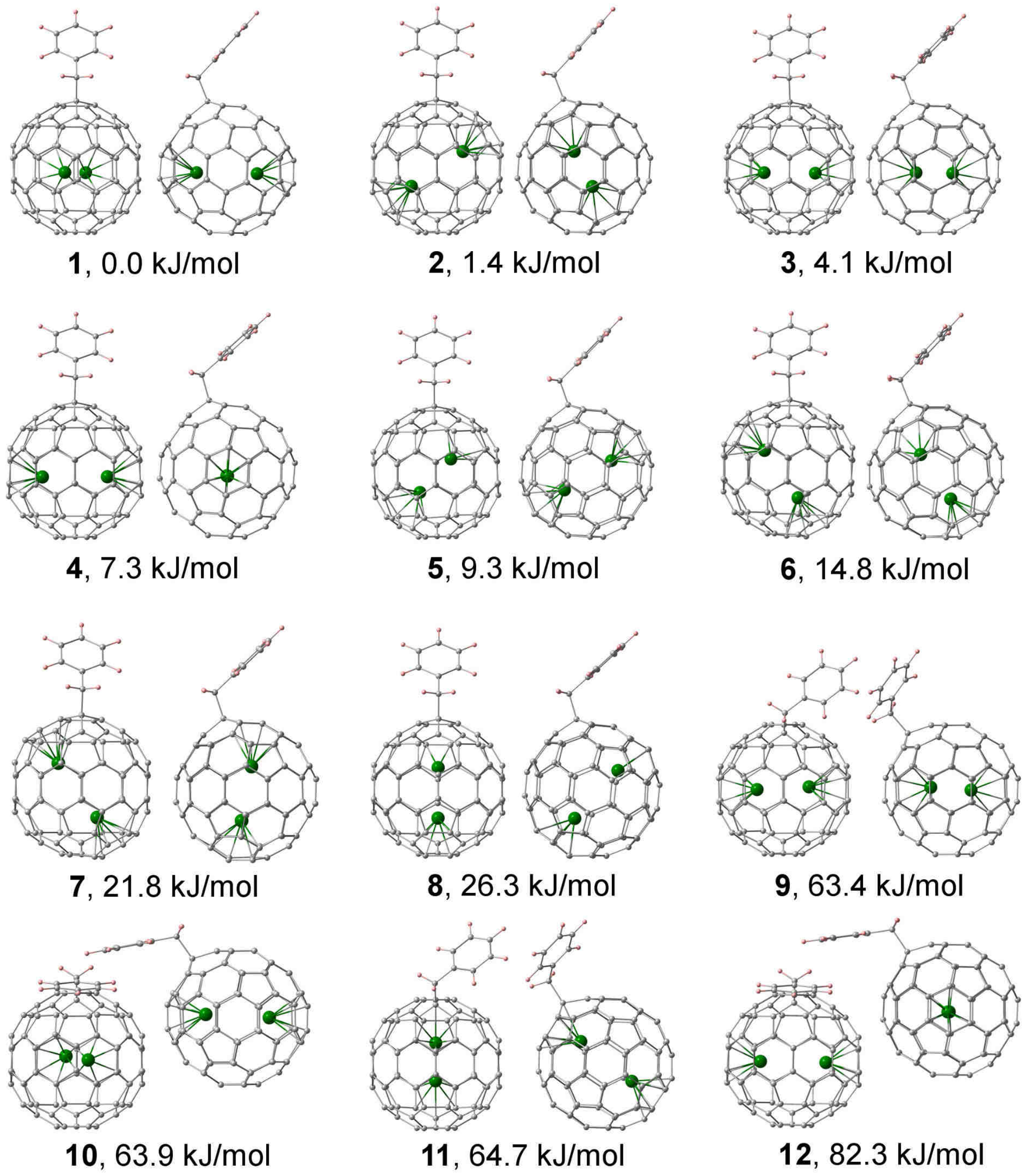

Supplementary Figure 14. DFT-optimized molecular structures and relative energies of $Y_2@C_{80}(CH_2Ph)$ structures. Calculations are performed at the PBE/TZ2P level. Each structure is shown in two projections. Structures **9**–**12** have CH_2Ph groups on the triple-hexagon junction, in all others CH_2Ph is added to the pentagon/hexagon/hexagon junction. The lowest energy structure **1** has the same position of metal atoms as in the X-ray structure of **Dy_2-I**.

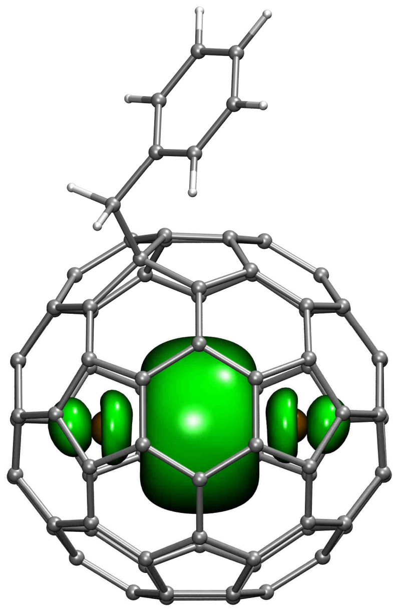

Supplementary Figure 15. Spin density distribution in **Y_2-I.**

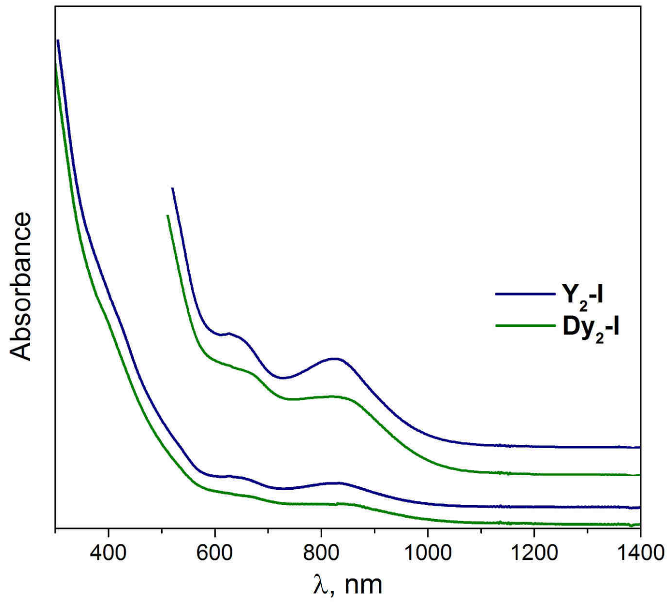

Supplementary Figure 16. UV-vis-NIR absorption spectra of **Y_2-I** and **Dy_2-I** in toluene solution. The low-energy range is additionally enhanced.

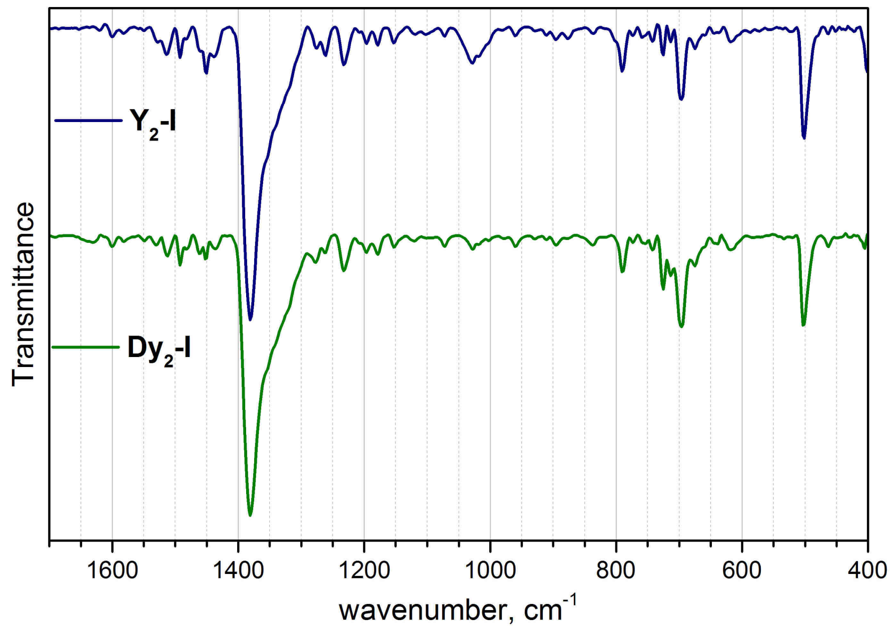

Supplementary Figure 17. Infrared spectra of **Y_2-I** and **Dy_2-I** drop-casted on KBr.

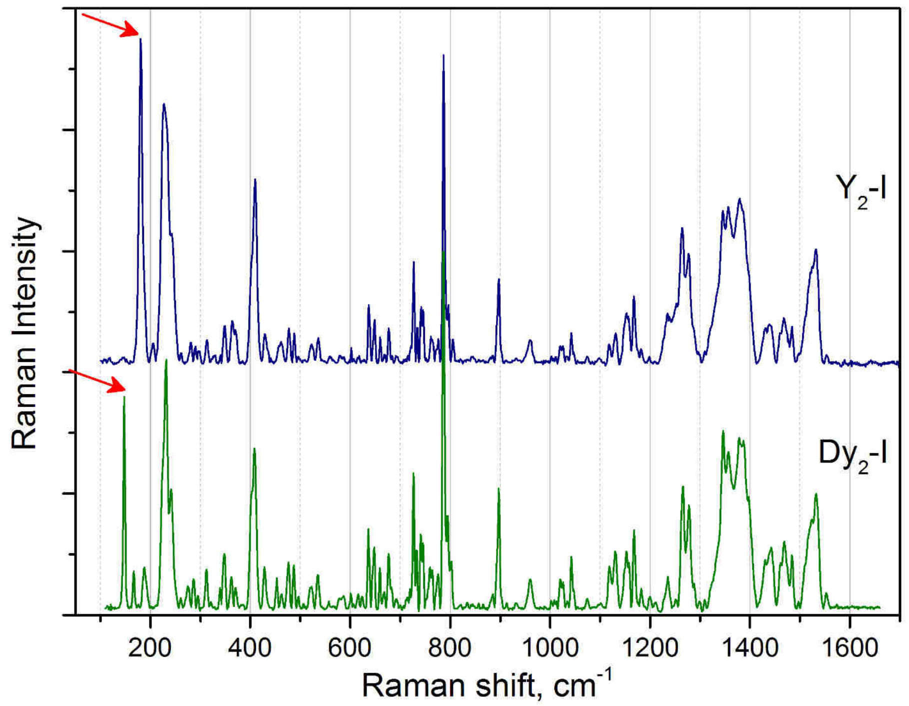

Supplementary Figure 18. Raman spectra of **Y_2-I** and **Dy_2-I** drop-casted on KBr and measured at 78 K. Red arrows mark the peaks with strong metal dependence, assigned to the "metal-cage stretching" vibration at 148 cm^{-1} in **Dy_2-I** and 181 cm^{-1} in **Y_2-I**.

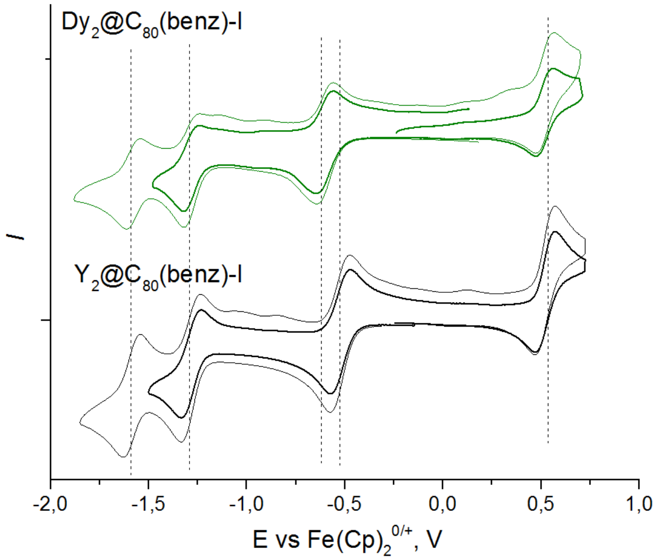

Supplementary Figure 19. Cyclic voltammetry of **Dy_2-I** and **Y_2-I** in o-dichlorobenzene at the sweep rate of 100 mV/s. Three reversible reduction steps can be well seen. However, appearance of additional features in the back scan is detected if potential window includes the third reduction step. This points to the limited stability of the trianion in solution.

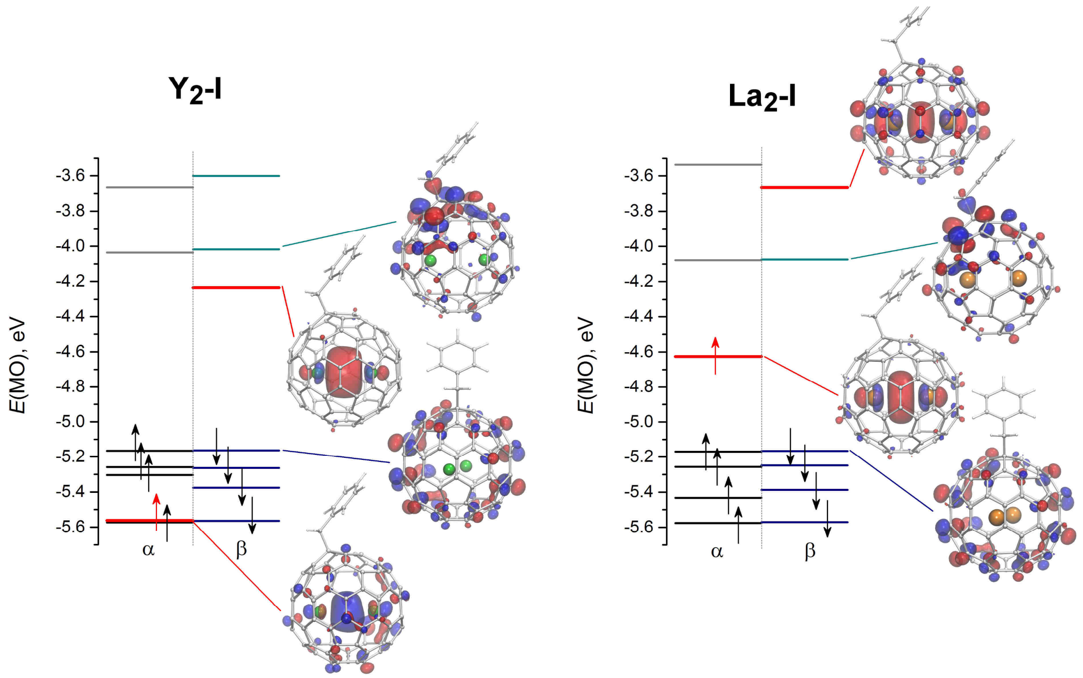

Supplementary Figure 20. Molecular orbital energies in **Y_2-I** and **La_2-I**. Metal-based levels are highlighted in red. Note that in **Y_2-I**, HOMO levels are fullerene-based, whereas the lowest unoccupied MO is localized on the Y_2-fragment. In **La_2-I**, the highest occupied level is localized on La_2-dimer, whereas LUMO levels are localized on the fullerene core. Computations are perfumed at the PBE-ZORA/TZVP level.

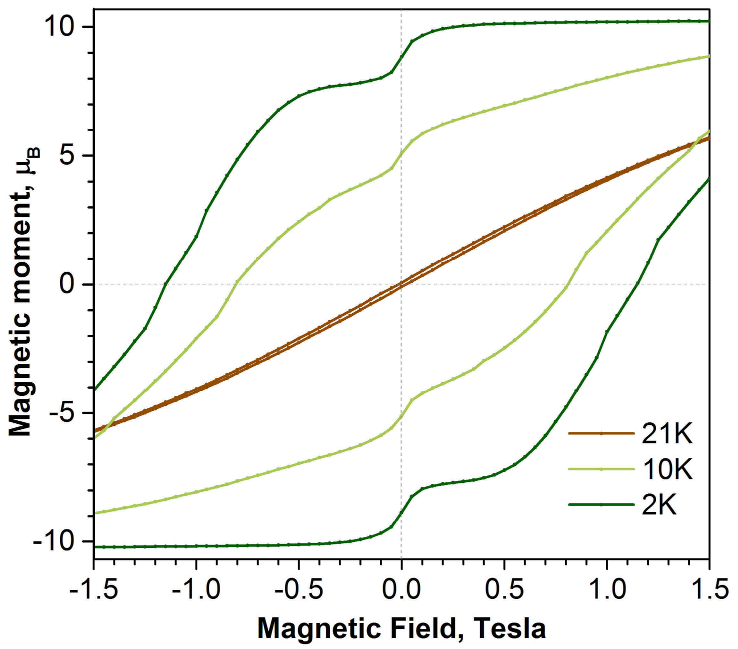

Supplementary Figure 21. Magnetization curves of **Dy_2-I**. Curves are measured at 2, 10, and 21 K with the field sweep rate of 2.9 mT/s. Hysteresis in the magnetization curves of **Dy_2-I** is observed up to the temperature of 21 K. At 22 K, the hysteresis closes.

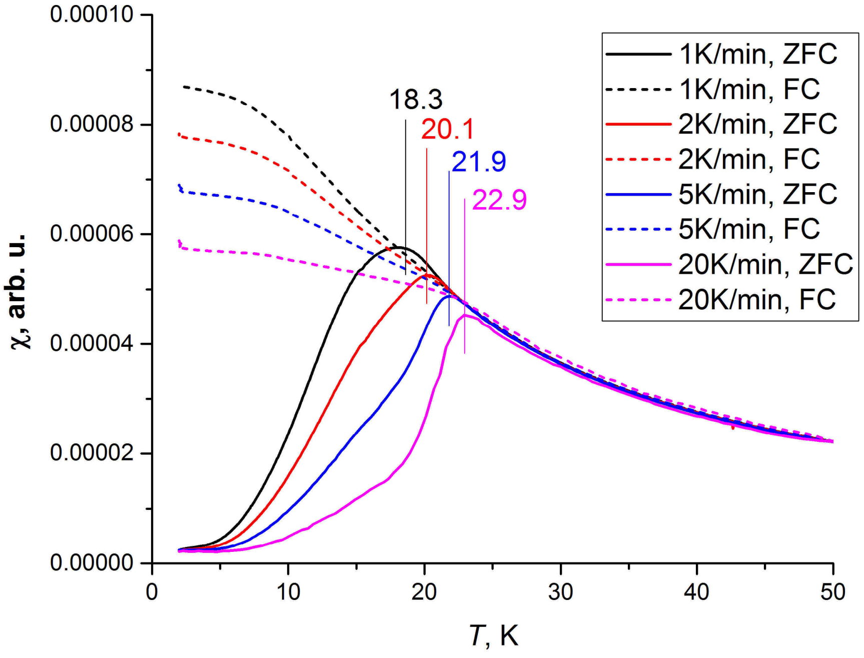

Supplementary Figure 22. Determination of blocking temperature T_B. The sample is first cooled in zero-field (ZFC) to 1.8 K, then χ is measured in the field of 0.2 T with increasing temperature (solid curve), then the measurement is performed at cooling down to 1.8 K (dashed curve). The vertical bars denotes T_B values determined with different temperature sweep rate.

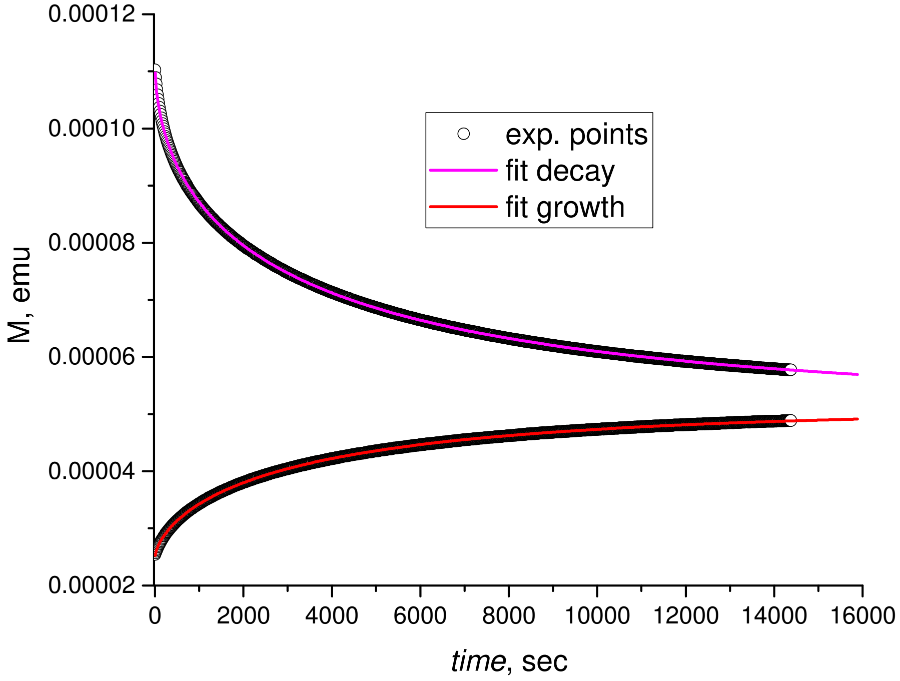

Supplementary Figure 23. Determination of magnetization relaxation time from the magnetization decay curve. For the measurement at 0.4 T, magnetization does not decay to zero. To determine equilibrium magnetization (y_0), the second measurement, growth of magnetization for a zero-field sample, was used and fitted together with the decay curve. The figure shows the curves measured for *T* = 7 K. Dots are experimental data, lines are fits with stretched exponential function.

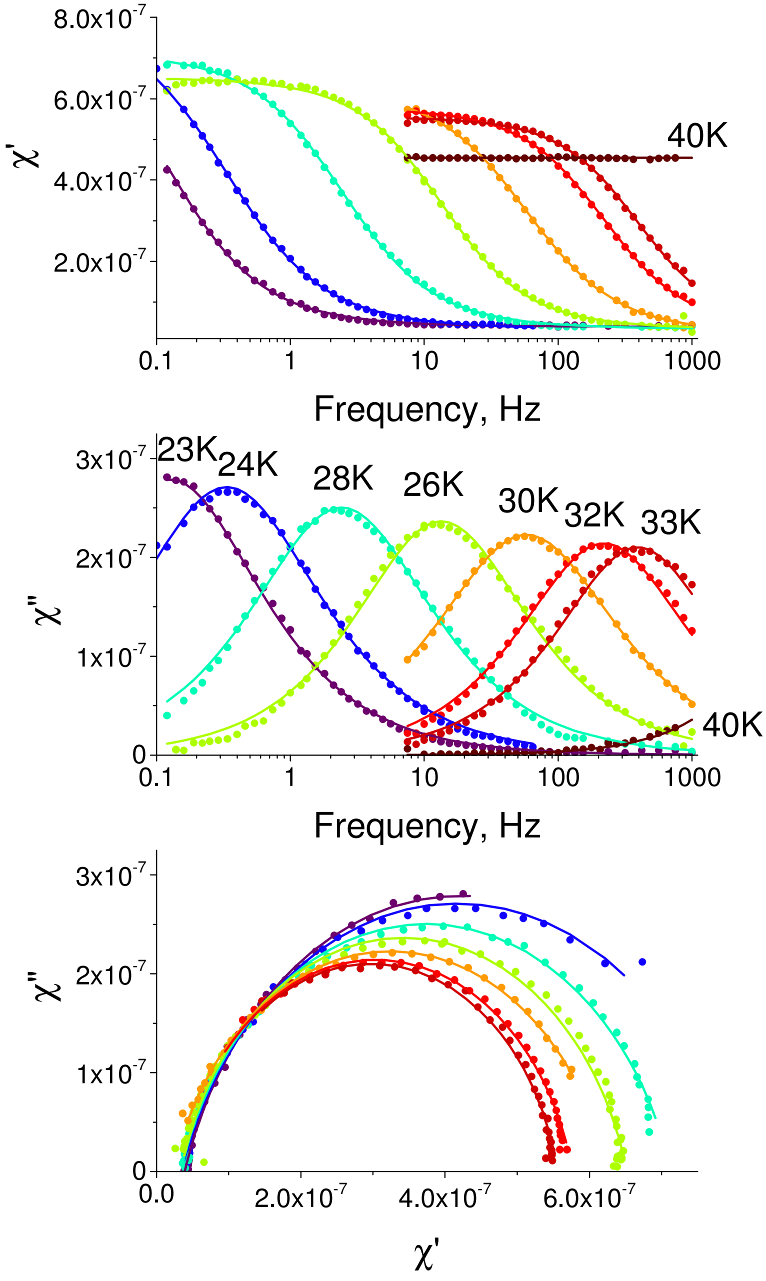

Supplementary Figure 24. Ac-magnetic susceptibility measurements. The plot show in-phase (top), out-of-phase (middle) susceptibilities as well as Cole-Cole plots (bottom). Dots are experimental plots, lines are fitting with generalized Debye model.

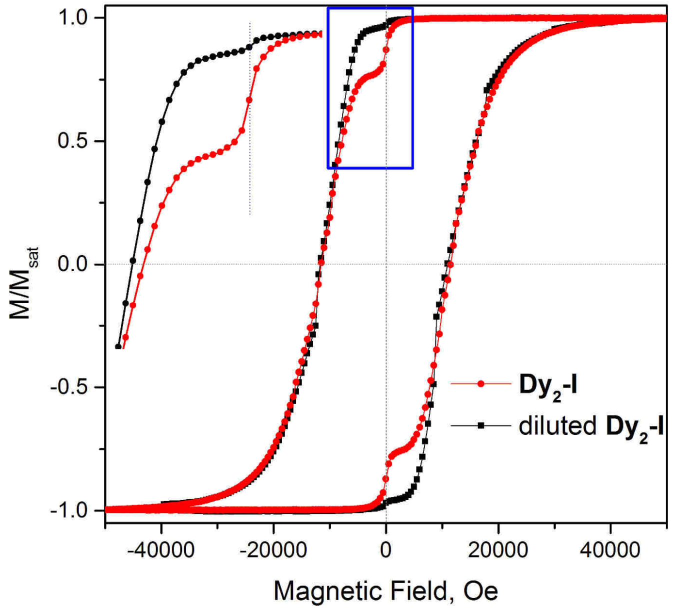

Supplementary Figure 25. Magnetization measurement of the powder **Dy$_2$-I** and the sample dispersed in polystyrene at 2 K. Red points are measurements for the bulk sample, black points are measured for the samples dispersed in polystyrene (ca 1:10,000 mass ratio; black points). Diamagnetic background of polystyrene is subtracted. The part of the curve near zero field is enhanced. The drop of magnetization near zero-field is assigned to the temperature-independent QTM-like process. In the diluted sample, the drop is dramatically reduced, and relaxation times become substantially longer.

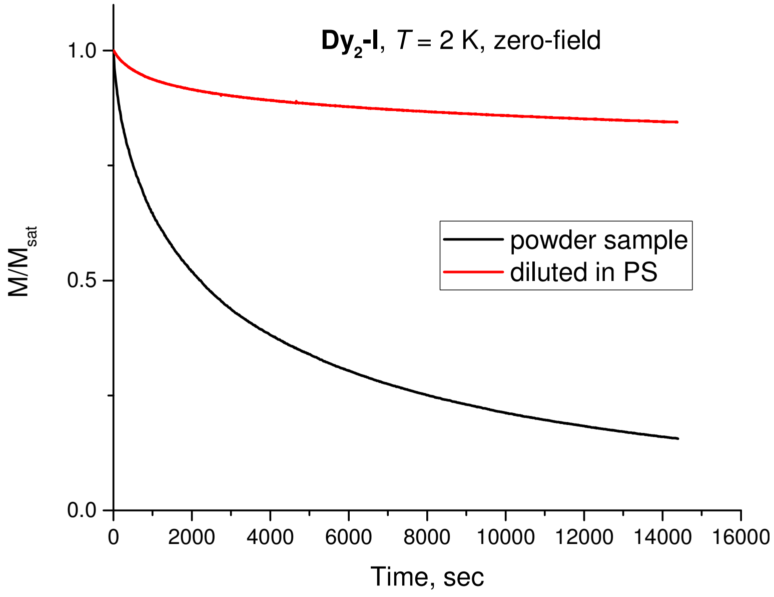

Supplementary Figure 26. Typical magnetization relaxation curves measured for **Dy$_2$-I** as a powder and as dispersed in polystyrene.

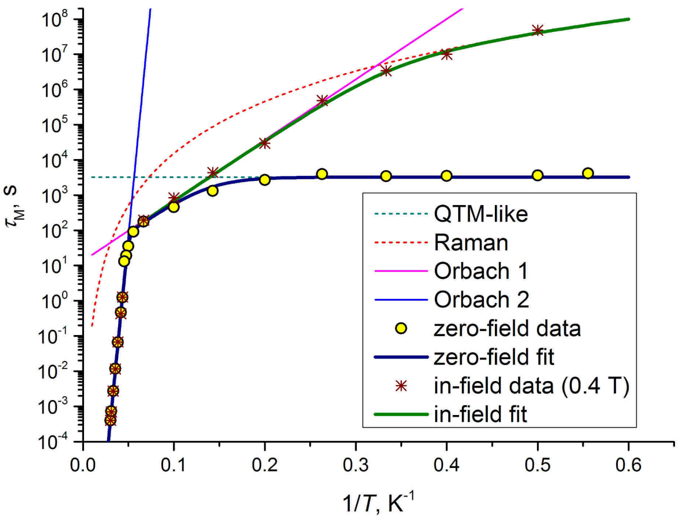

Supplementary Figure 27. Magnetization relaxation times of **Dy_2-I.** The points are measured in the field of 0.4 T and in zero-field. The lines are fits with the equations (1) and (2) and contribution of individual mechanisms (Raman, two Orbach processes, and QTM-like temperature-independent regime, which is available only for zero-field measurements). Note that ac-measurements gave virtually identical values for zero-field and the field of 0.4 T.

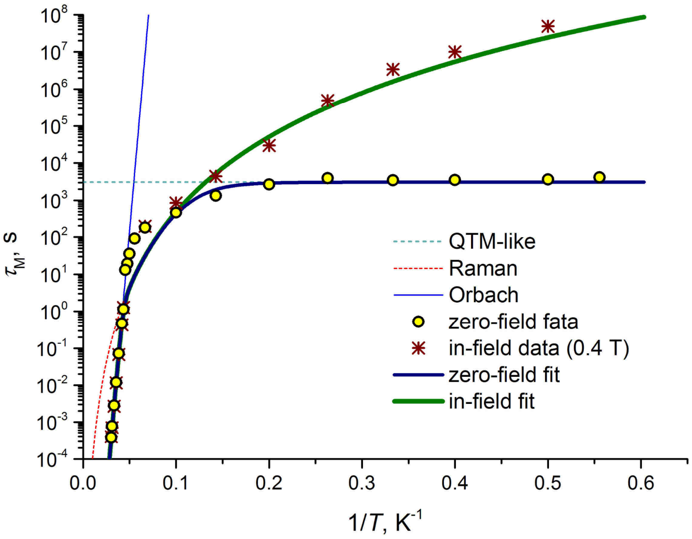

Supplementary Figure 28. Magnetization relaxation times of **Dy_2-I.** The points are measured in the field of 0.4 T and in zero-field. The lines are fits with the Supplementary Equations (1) and (2) and contribution of individual mechanisms (Raman, Orbach processes, and QTM-like temperature-independent regime, which is available only for zero-field measurements).

Dy1

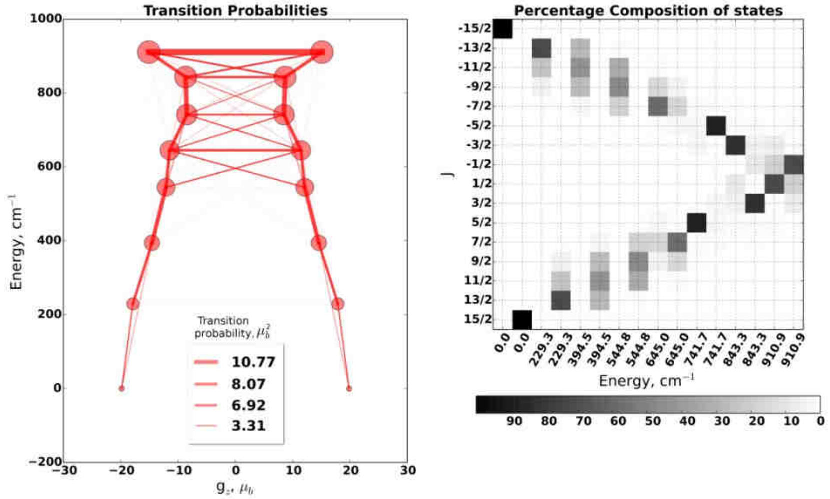

Dy2

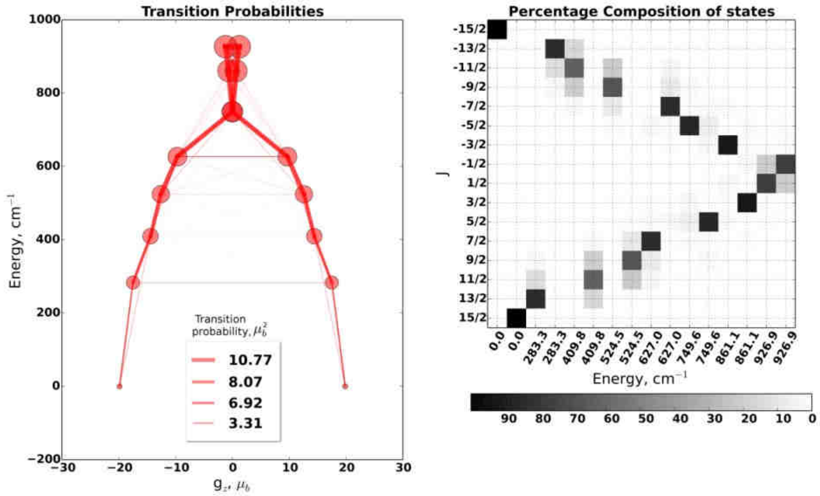

Supplementary Figure 29. Zero-field average transition probability between KD-sates for Dy1 and Dy2. The thickness of the red lines between two KD states is proportional to the transition probability between those states. Computations are performed using PHI code, probability of transition between the state *i* and *f* is computed as $T_{if} = \frac{1}{3}\sum_{\alpha=x,y,z}\left|\langle i\left|\hat{H}_Z(B_\alpha)\right|f\rangle\right|^2$

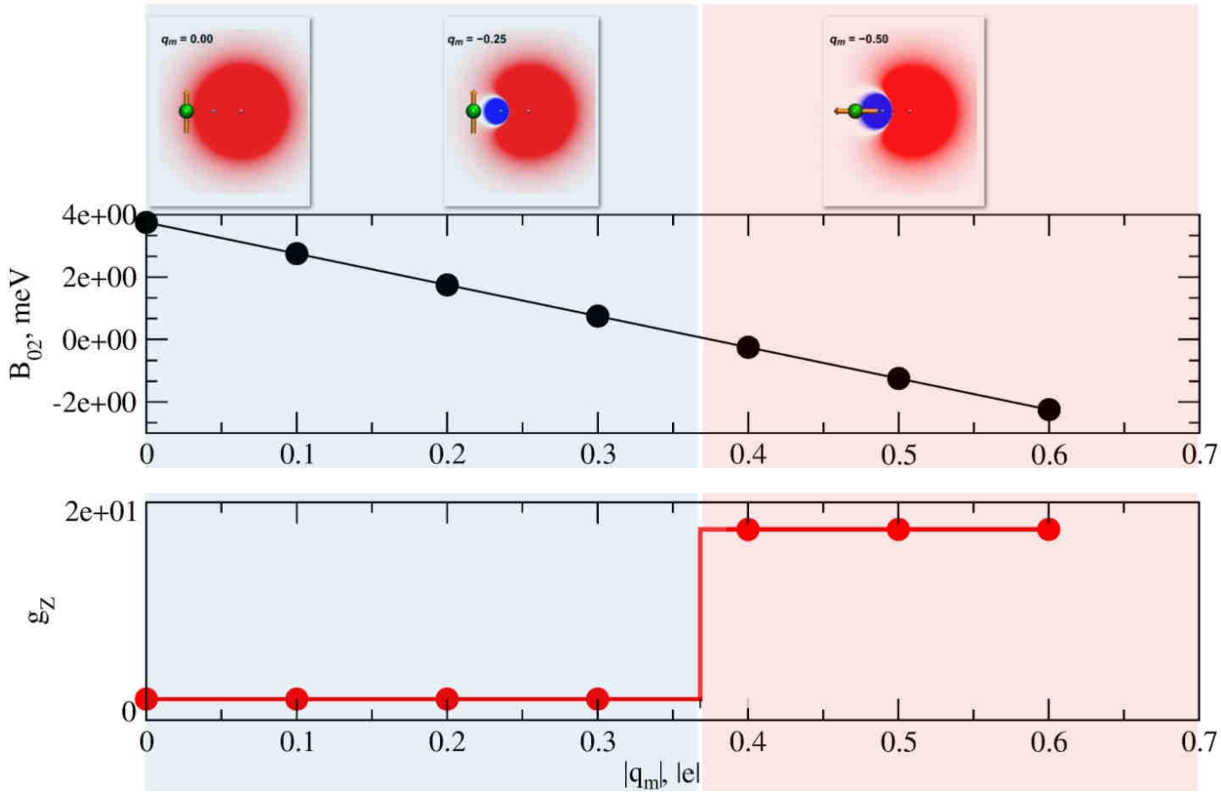

Supplementary Figure 30. Systematic change of the midpoint charge (q_m) in the model system [Dy^{3+}–q_m–(+3)]. The main CF-parameter B_0^2 changes continuously, while g_Z projection changes discontinuously at a critical value q_m=−0.36

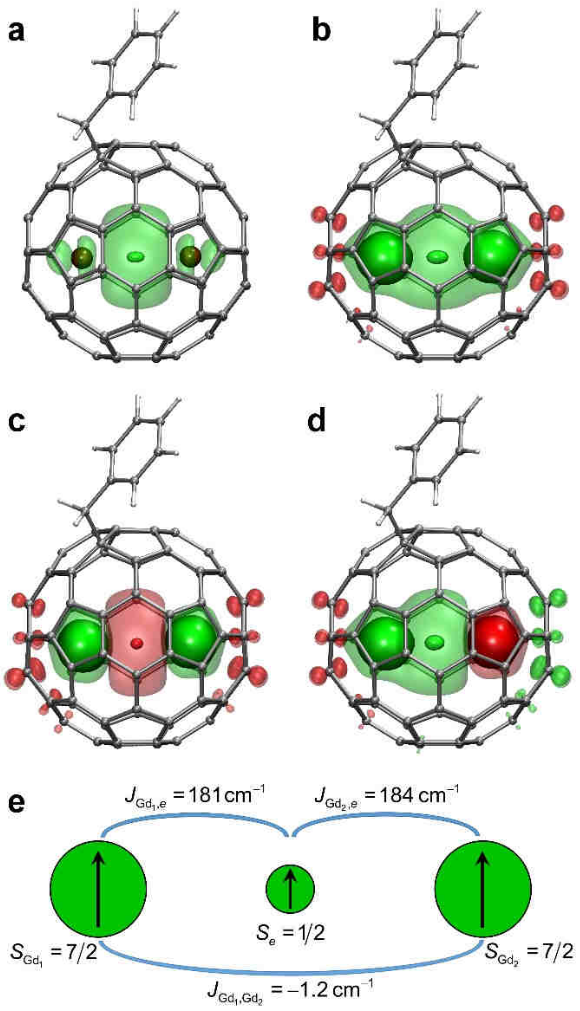

Supplementary Figure 31. Spin density and exchange interactions in **Gd_2-I**. (**a**) spin density in **Y_2-I**; (**b**) spin density in the high-spin ground state of **Gd_2-I**; (**c,d**) broken-symmetry states of **Gd_2-I** with the spin flipped at the single electron center (**c**) or at one of the Gd centers (**d**). Spin density isosurfaces are shown with two isovalues, 0.015 a.u. (solid) and 0.0012 a.u. (transparent), green and red denote positive and negative values of the spin density, respectively. (**e**) Schematic depiction of the exchange interactions in **Gd_2-I**, described as a 3-center spin system; exchange parameters are obtained by mapping the energies of broken-symmetry states on the Hamiltonian in Supplementary Equation (3).

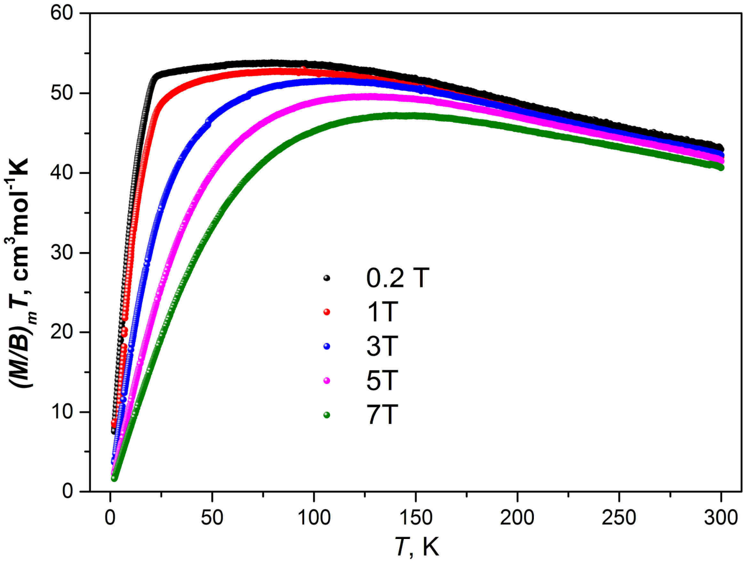

Supplementary Figure 32. *(M/B)T* function measured for **Dy_2-I** in different magnetic fields from 0.2 T to 7 T. Note that the sharp feature in the curve measured at 0.2 T corresponds to the blocking temperature of the compound. At lower temperatures, the values deviate significantly from the thermodynamic limit and hence cannot be used to compare with simulated curves (which necessary correspond to the equilibrium situation).

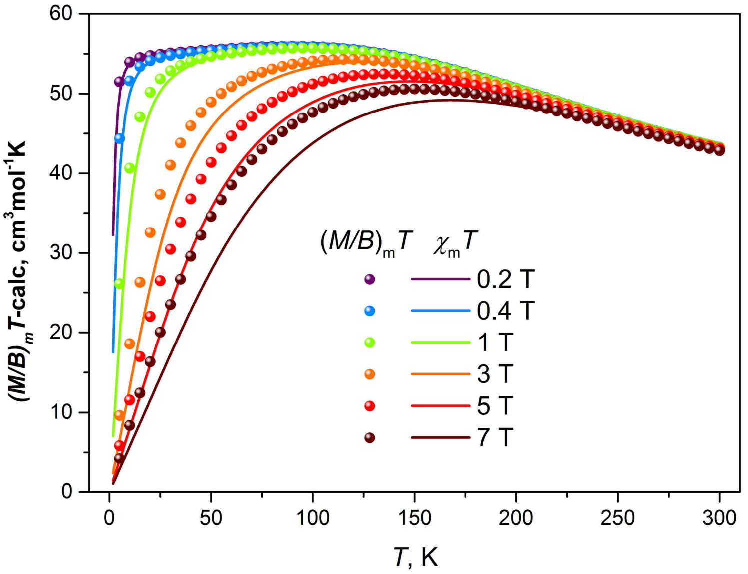

Supplementary Figure 33. Comparison between *(M/B)T* and *χT* functions. Curves are computed for **Dy_2-I** with different values of the magnetic field B. Computations were performed for the Hamiltonian in Equation (4), in which CF parameters were computed *ab initio* as discussed above, and $j_{Dy,e}$ constant is set to 32 cm^{-1}.

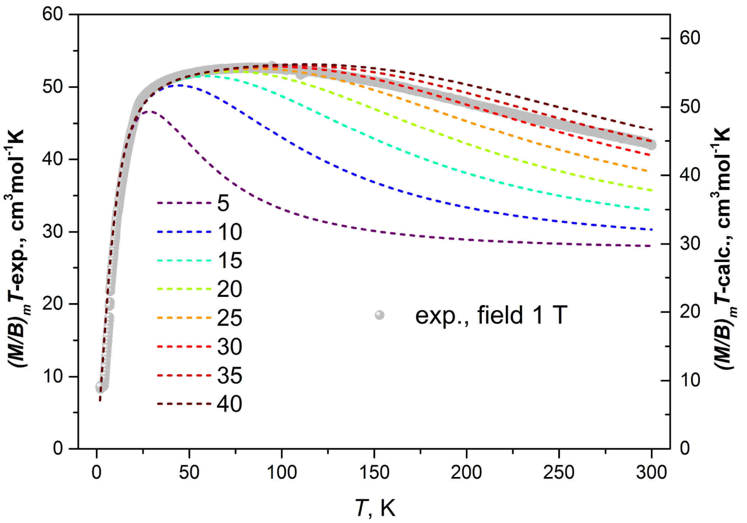

Supplementary Figure 34. Comparison between the *(M/B)*$_m$*T* function measured at 1 T and simulated χ_mT functions. Simulations are performed for **Dy$_2$-I** with different values of the $j_{\mathrm{Dy},e}$ parameter (the values are given in cm^{-1}) and CASSCF-computed CF parameters.

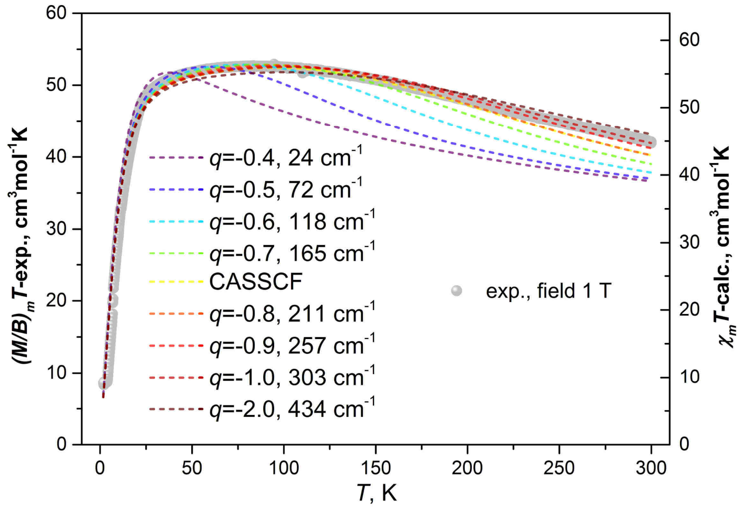

Supplementary Figure 35. Comparison between the *(M/B)*$_m$*T* function measured at 1 T and simulated χ_mT functions. Computations are performed for **Dy$_2$-I** with different CF parameters (for each set of parameters, shown are the point negative charge and the energy difference between the ground and the first excited state in cm^{-1}) and the $j_{\mathrm{Dy},e}$ constant of 30 cm^{-1}.

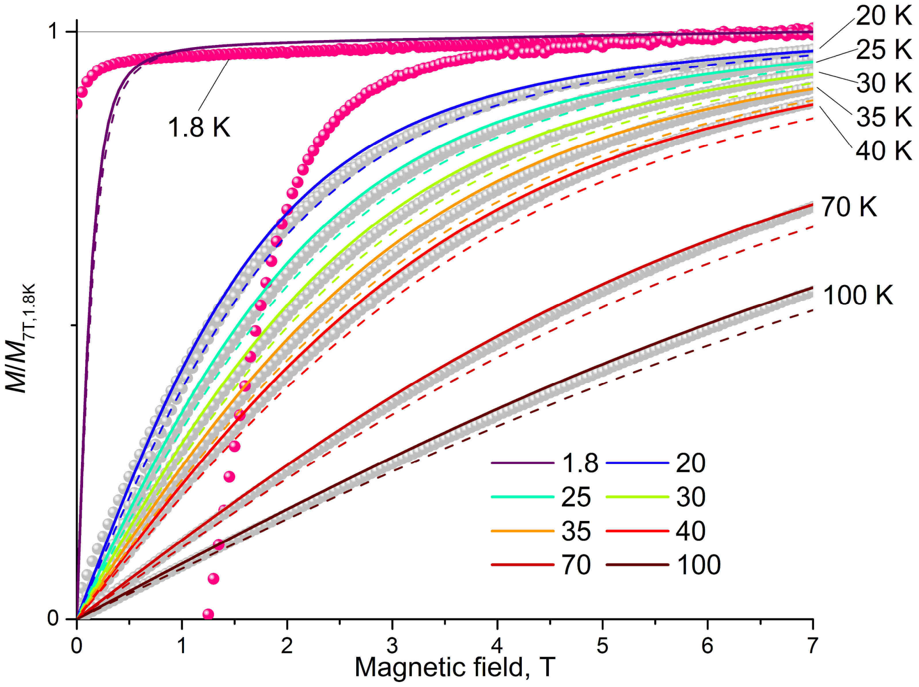

Supplementary Figure 36. Normalized magnetization curves of **Dy_2-I** measured at different temperatures. Dots are experimental values, lines are simulations for the ferromagnetic (solid lines) and antiferromagnetic (dashed lines) coupling in the [Dy^{3+}–*e*–Dy^{3+}] system.

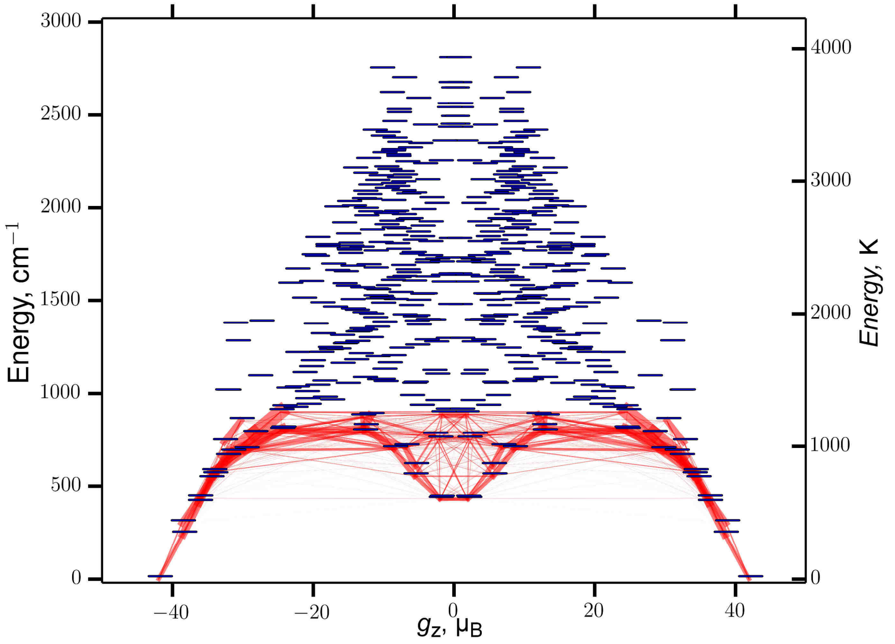

Supplementary Figure 37. The spectrum of the effective spin Hamiltonian. Transition probabilities for the low-energy range are visualized as lines of different thickness (thicker lines correspond to higher probabilities).

Supplementary Table 1. Crystal data and data collection parameters

Crystal	$Dy_2@I_h(7)\text{-}C_{80}\text{-}CH_2C_6H_5/0.67(C_7H_8)$
Formula	$C_{91.66}\ H_{12.33}\ Dy_2$
Formula weight	1438.27
Color, habit	Black, block
Crystal system	triclinic
Space group	*P-1*
a, Å	10.890(2)
b, Å	11.040(2)
c, Å	19.300(4)
α, deg	85.91(3)
β, deg	88.89(3)
γ, deg	78.06(3)
Volume, $Å^3$	2264.3(8)
Z	2
T, K	100
Radiation (λ, Å)	Synchrotron Radiation (0.89429)
Unique data (R_{int})	9024 (0.055)
Parameters	857
Restraints	770
Observed data ($I > 2\sigma(I)$)	7591
$R1^a$ (observed data)	0.0792
$wR2^b$ (all data)	0.2570

[a]For data with $I > 2\sigma(I)$, $R1=1\|F_O|-|F_C\|/\Sigma|F_O|$. [b]For all data, $wR2 =\{\Sigma[w(F_O^2-F_C^2)^2])/\Sigma[w(F_O^2)^2]\}^{1/2}$.

Supplementary Table 2. Magnetization relaxation times determined from the stretched exponential fitting of zero-field DC relaxation measurements.

T, K	τ, sec	St. dev., sec	β
1.8	4157.11	6.26	0.53
2	3658.87	8.58	0.51
2.5	3502.90	7.08	0.51
3	3456.12	5.24	0.52
3.8	3936.77	5.20	0.53
5	2691.35	1.31	0.62
7	1327.86	0.99	0.75
10	455.37	0.41	0.77
15	179.80	0.41	0.80
18	91.56	0.21	0.86
20	35.86	0.13	0.89
21	19.22	0.06	0.85
22	13.12	0.25	0.74

Supplementary Table 3. Magnetization relaxation times determined from the stretched exponential fitting of 0.4 T DC relaxation measurements.

T, K	τ, sec	St. dev., sec	β
2	4.8510E+07	6.0780E+06	0.50
2.5	1.0062E+07	4.9340E+05	0.53
3	3.4072E+06	8.9052E+04	0.52
3.8	4.8518E+05	1.3001E+03	0.58
5	29799.67	320.46	0.67
7	4381.83	4.07	0.73
10	843.58	0.40	0.79
15	195.79	0.35	0.82

Supplementary Table 4. Relaxation times determined from zero-field AC measurements by fitting to a generalized Debye model

T, K	τ, sec	St. dev., sec	α
23	1.14	0.025	0.20
24	0.47	0.006	0.22
26	7.23E-02	5.18E-04	0.19
28	1.20E-02	1.12E-04	0.17
30	2.83E-03	1.68E-05	0.19
32	7.76E-04	6.93E-06	0.15
33	3.77E-04	5.67E-06	0.13

Supplementary Table 5. Relaxation times determined from 0.4 T-field AC measurements by fitting to a generalized Debye model

T, K	τ, sec	St. dev., sec	α
23	1.269	0.037	0.19
24	0.430	0.008	0.20
26	6.76E-02	6.96E-04	0.20
28	1.16E-02	1.69E-04	0.18
30	2.70E-03	2.43E-05	0.19
32	7.03E-04	9.72E-06	0.16
33	4.03E-04	6.84E-06	0.13

Supplementary Table 6. *CASSCF/SO-RASSI/SINGLE_ANISO calculations results summary for the [**DyY-I**]$^{-}$ molecule. G-tensors components and energies of the eight low lying KDs for the Dy1 and Dy2 ions.*

Kramers Doublet	**[DyY-I]$^{-}$,Dy$_1$**		**[DyY-I]$^{-}$,Dy$_2$**	
#	state energy, cm^{-1}	g_x | g_y |g_z, μ_B	state energy, cm^{-1}	g_x | g_y |g_z, μ_B
KD1	0.0	0.00|0.00|19.83	0.0	0.00|0.00|19.86
KD2	227.6	0.01|0.01|17.88	281.8	0.03|0.03|17.50
KD3	393.1	0.12|0.14|14.55	410.5	0.19|0.21|14.41
KD4	545.0	0.67|0.92|12.10	525.9	0.87|1.34|12.61
KD5	643.5	1.36|2.76|11.47	625.7	1.89|3.96|9.69
KD6	737.6	1.04|4.48|8.48	746.9	7.03|5.52|0.00
KD7	843.2	0.84|3.36|8.66	861.5	5.89|3.60|0.75
KD8	913.0	1.04|5.68|15.07	929.2	13.1|7.85|1.20

Supplementary Table 7. *Ab-intio derived (SINGLE_ANISO) crystal field parameters in Stevens Notation B(q,k)(cm^{-1}) for Dy-ions in the* [**DyY-I**]$^{-}$ *molecule and model systems.*

index		**[DyY-I]$^{-}$, Dy$_1$**	**[DyY-I]$^{-}$, Dy$_2$**
**k**	**q**	**B(q,k)**	**B(q,k)**
2	-2	6.02E-02	-2.23E-01
2	-1	1.51E+00	3.18E+00
2	0	-4.95E+00	-4.93E+00
2	1	-1.27E+00	-7.77E-01
2	2	8.38E-01	7.48E-01
4	-4	-1.43E-04	1.29E-04
4	-3	-2.46E-03	-7.38E-03
4	-2	5.46E-04	7.16E-05
4	-1	-6.90E-04	-3.41E-03
4	0	9.24E-04	8.49E-04
4	1	9.96E-04	1.03E-04
4	2	3.39E-03	3.96E-03
4	3	8.85E-04	3.31E-04
4	4	-7.84E-04	-1.00E-03
6	-6	1.08E-04	-8.48E-05
6	-5	8.33E-04	7.29E-04
6	-4	-1.16E-05	1.04E-04
6	-3	-1.29E-04	-1.41E-04
6	-2	2.34E-06	3.04E-05
6	-1	-1.83E-04	-3.77E-04
6	0	-4.73E-05	-3.64E-05
6	1	1.35E-04	7.69E-05
6	2	-1.18E-04	-2.88E-05
6	3	-2.91E-05	-1.44E-04
6	4	-1.39E-04	-1.37E-04
6	5	2.82E-04	9.00E-04
6	6	1.95E-04	1.33E-04

**[Dy-Y]$^{+6}$**	**[Dy-Y]$^{+4}$**	**[Dy-H-Y]$^{+5}$**
**B(q,k)**	**B(q,k)**	**B(q,k)**
1.17E-07	-4.62E-06	7.66E-05
-3.97E-08	-4.38E-07	2.78E-05
3.99E+00	-5.36E+00	-3.93E+00
5.84E-07	-4.66E-07	-4.56E-08
-3.18E-07	9.45E-06	-5.45E-06
1.69E-10	6.05E-09	6.51E-05
3.47E-09	-6.17E-08	1.34E-07
1.76E-09	-1.28E-08	-6.82E-07
5.81E-11	-8.58E-09	-4.62E-07
6.92E-04	-1.03E-03	-2.48E-03
3.75E-10	-8.13E-10	-1.49E-09
9.66E-10	2.68E-09	-1.53E-07
-2.76E-09	-3.90E-08	-2.89E-07
-2.72E-09	3.32E-09	4.67E-05
1.55E-11	-3.70E-12	-1.69E-08
1.52E-11	5.52E-12	1.43E-09
4.13E-13	-3.86E-11	3.58E-06
2.21E-11	2.25E-10	-1.20E-09
-1.28E-11	3.27E-10	-4.27E-09
1.13E-11	2.23E-10	8.08E-09
-4.87E-07	4.57E-06	2.10E-05
-3.34E-12	8.31E-11	6.83E-11
3.36E-12	-3.38E-10	-1.00E-09
1.74E-11	1.24E-10	3.93E-09
5.14E-12	-1.12E-11	-1.07E-07
-1.34E-10	-1.03E-11	2.41E-09
6.75E-12	-1.76E-12	-2.33E-08

Supplementary Table 8. *Point charge model calculations results summary for a set of model systems (homotopic to atomistic systems in* **Table S4**, *i.e.* **[Dy-Y]$^{+6}$ ~ [Dy-(+3)], [Dy-Y]$^{+4}$ ~ [Dy–(−1)–(+3)]** *and* **[Dy–(−0.5)–(+3)]~ [Dy–H–Y]$^{+5}$**). *G-tensors components and energies of the eight low lying KD of the Dy center.*

KD	[Dy–(+3)]		[Dy–(−1)–(+3)]			[Dy–(−0.5)–(+3)]	
J	E, cm^{-1}	g_x | g_y |g_z, μb	J	E, cm^{-1}	g_x | g_y |g_z, μb	E, cm^{-1}	g_x | g_y |g_z, μb
KD1	0.0	10.7|10.7 |1.33	KD1	0.0	0.00|0.00 |20.00	0.0	0.00|0.00 |20.00
KD2	21.5	0.00|0.00 |4.00	KD2	308.3	0.00|0.00 |17.33	71.1	0.00|0.00 |17.33
KD3	64.8	0.00|0.00 |6.67	KD3	565.9	0.00|0.00 |14.67	130.1	0.00|0.00 |14.67
KD4	130.6	0.00|0.00 |9.33	KD4	761.9	0.00|0.00 |12.00	171.1	0.00|0.00 |12.00
KD5	219.7	0.00|0.00 |12.00	KD5	898.9	0.00|0.00 |9.33	194.9	0.00|0.00 |9.33
KD6	333.0	0.00|0.00 |14.67	KD6	986.6	0.00|0.00 |6.67	205.7	0.00|0.00 |6.67
KD7	471.7	0.00|0.00 |17.33	KD7	1037.0	0.00|0.00 |4.00	209.0	0.00|0.00 |4.00
KD8	636.6	0.00|0.00 |20.00	KD8	1059.0	10.7 |10.7|1.33	209.4	10.7 |10.7|1.33

Supplementary Table 9. *CASSCF/SO-RASSI/SINGLE_ANISO calculations results summary for a set of model systems (Two metal dimers in a different oxidation state,* **[Dy-Y]$^{+6}$** and **[Dy-Y]$^{+4}$**, *as well as and* **[Dy-Y]$^{+4}$** *with a proton placed at the midpoint giving* **[Dy-H-Y]$^{+5}$**). *G-tensors components and energies of the eight low lying KD of the Dy center.*

KD	[Dy-Y]$^{+6}$			[Dy-Y]$^{+4}$		[Dy-H-Y]$^{+5}$	
	E, cm^{-1}	g_x | g_y |g_z, μb		E, cm^{-1}	g_x | g_y |g_z, μb	E, cm^{-1}	g_x | g_y |g_z, μb
KD1	0.0	10.7|10.7|1.35	KD1	0.0	0.00|0.00|19.9	0.0	0.00|0.00|19.9
KD2	21.13	0.00|0.00|4.03	KD2	236.3	0.00|0.00|17.1	164.1	0.00|0.00|17.1
KD3	64.16	0.00|0.00|6.72	KD3	441.3	0.00|0.00|14.4	349.5	0.00|0.00|14.4
KD4	130.5	0.00|0.00|9.40	KD4	607.1	0.00|0.00|11.7	496.2	0.00|0.00|11.8
KD5	222.3	0.00|0.00|12.1	KD5	731.6	0.00|0.00|9.14	580.7	0.00|0.00|9.20
KD6	341.8	0.00|0.00|14.7	KD6	817.8	0.00|0.00|6.53	610.7	0.28|0.28|6.62
KD7	491.5	0.00|0.00|17.3	KD7	870.8	0.00|0.00|3.92	628.4	10.6|10.6|1.35
KD8	672.9	0.00|0.00|19.9	KD8	895.8	10.7|10.7|1.31	641.3	0.28|0.29|3.98

Note that **[Dy-Y]$^{+6}$** system gives easy-plane magnetic anisotropy for Dy ion, whereas **[Dy-Y]$^{+4}$** and **[Dy-H-Y]$^{+5}$** enable easy-axis anisotropy.

Supplementary Table 10. *Point charge model (MCPhase) crystal field parameters in the Stevens notation B(q,k)(cm^{-1}) for model systems.*

index		[Dy–(+3)]	[Dy–(−1)–(+3)]	[Dy–(−0.5)–(+3)]
**k**	**q**	**B(q,k)**	**B(q,k)**	**B(q,k)**
2	0	3.87E+00	-6.25E+00	-1.25E+00
2	4	3.10E-04	-2.85E-03	-1.28E-03
2	6	-1.94E-07	7.55E-06	3.69E-06

Supplementary Table 11. Energies and composition of the lowest energy KD states of the [Dy^{3+}–*e*–Dy^{3+}] system computed with $j_{Dy,Dy}$ = 0.0 cm^{-1} and $j_{Dy,e}$ = 32 cm^{-1}

<table>
<tr><td>E, cm^{−1}</td><td>Composition in terms of J_z states of individual centers [a]</td><td></td></tr>
<tr><td>0.0</td><td>99.4% |−15/2,−15/2,−1/2></td><td></td></tr>
<tr><td>241</td><td>66.6% |−13/2,−15/2,−1/2> + 15.4% |−11/2,−15/2,−1/2>+ 8.1% |−15/2,−13/2,−1/2></td><td></td></tr>
<tr><td>302</td><td>75.5% |−15/2,−13/2,−1/2> + 9.2% |−15/2,−11/2,−1/2>+ 5.9% |−13/2,−15/2,−1/2>+</td><td></td></tr>
<tr><td>412</td><td>37.4% |−11/2,−15/2,−1/2> + 17.3% |−9/2,−15/2,−1/2> + 9.3% |−15/2,−11/2,−1/2> +
18.1% |−13/2,−15/2,−1/2></td><td></td></tr>
<tr><td>428</td><td>63.3% |−15/2,+15/2,+1/2> + 17.3% |+15/2,−15/2,−1/2> + 10.0% |−13/2,+15/2,−1/2></td><td></td></tr>
<tr><td>434</td><td>84.9% |−15/2,+15/2,−1/2> + 10.8% |−13/2,+15/2,+1/2></td><td></td></tr>
<tr><td>436</td><td>52.5% |−15/2,−11/2,−1/2> + 12.4% |−15/2,−9/2,−1/2> + 11.2% |−15/2,−13/2,−1/2></td><td></td></tr>
</table>

[a] Only one component of each doublet state is shown, composition of another one is obtained by changing the sign of all J_z components

Supplementary Table 12. The energies and g-tensors for the lowest energy states of the [Dy^{3+}–*e*–Dy^{3+}] system

---

g-tensor of states 1 and 2; Energy 0.0 cm^{-1}

		x	y	z
g_x =	0.0000	-0.99998575	0.00529543	-0.00067906
g_y =	0.0000	-0.00529686	-0.99998371	0.00212456
g_z =	41.9400	-0.00066780	0.00212813	0.99999751

---

g-tensor of states 3 and 4; Energies 240.6 cm^{-1}

		x	y	z
g_x =	0.0000	-0.99580500	0.08180580	0.04099050
g_y =	0.0000	-0.08796603	-0.97921306	-0.18276694
g_z =	39.1787	0.02518704	-0.18560601	0.98230139

---

g-tensor of states 5 and 6; Energies 301.8 cm^{-1}

		x	y	z
g_x =	0.0000	-0.98145402	-0.18895629	0.03230360
g_y =	0.0000	0.18238611	-0.97230855	-0.14612115
g_z =	39.1236	0.05901958	-0.13751946	0.98873914

---

g-tensor of states 7 and 8; Energies 412.2 cm^{-1}

		x	y	z
g_x =	0.0001	-0.99342249	0.10055374	0.05477865
g_y =	0.0001	-0.10544616	-0.98984955	-0.09528365
g_z =	36.4566	0.04464150	-0.10043312	0.99394181

---

g-tensor of states 9 and 10; Energies 428.2 cm^{-1}

		x	y	z
g_x =	0.0004	0.98759671	-0.15701049	-0.00066724
g_y =	0.0005	0.08673951	0.54912439	-0.83122720
g_z =	3.6169	0.13087779	0.82085938	0.55593245

---

g-tensor of states 11 and 12; Energies 434.3 cm^{-1}

		x	y	z
g_x =	0.0003	0.98445506	0.04660893	-0.16933944
g_y =	0.0079	-0.14739785	0.74353775	-0.65224649
g_z =	2.5337	0.09550976	0.66706763	0.73884956

---

g-tensor of states 13 and 14; Energy 436.2 cm^{-1}

		x	y	z
g_x =	0.0005	-0.86622479	0.49376868	0.07646632
g_y =	0.0009	-0.49673182	-0.86754222	-0.02505993
g_z =	36.3376	0.05396396	-0.05969079	0.99675719

----

g-tensor of states 15 and 16; Energy 537.7 cm^{-1}

		x	y	z
g_x =	0.0001	-0.99155522	0.05992866	0.11500781
g_y =	0.0002	-0.09562546	-0.93690899	-0.33623996
g_z =	37.0285	0.08760144	-0.34439817	0.93472771

Supplementary Note 1. Electron paramagnetic resonance (EPR) spectroscopy of Y-DMF extract and benzyl adducts

Supplementary Figure 2 shows the EPR spectrum of the Y-DMF extract in DMF at room temperature. The spectrum reveals the presence of several types of paramagnetic species. The strongest signal with g-factor of 2.0005 presumably corresponds to the anon radicals of empty fullerenes and/or oxidized forms of DMF. Besides, three triplet signals with hyperfine coupling constants of 65-75 G can be well seen. Their g-factors (determined from the position of the central peak in each triplet) are 1.9814, 1.9770, and 1.9744, and corresponding hfc constants are 64.5, 72.1, and 76.2 G. These combinations of g-factors and hfc constants result in coinciding position of the first peak of each of the three triplets. However, the second and the third components of the triplet signals are found at different fields. Due to the strong overlap, it is difficult to estimate the ratio of the peaks for each of the triplet. The ratio of the net integrals of the three triplets considered together is close to 1:2:1 as might be expected for $Y_2@C_{2n}^{-}$ anion radical with two equivalent ^{89}Y atoms (nuclear spin 1/2).

Reaction of $Y_2@C_{2n}^{-}$ anion radicals with benzyl bromide leads to a mixture of $Y_2@C_{2n}(CH_2Ph)$ derivatives. Note that each of the three major $Y_2@C_{2n}^{-}$ anions present in the DMF extract before the reaction may give several isomers of the benzyl adducts. Besides, Y atoms in such adducts may be non-equivalent. Combination of these two factors leads to a complex EPR spectrum of the mixture of $Y_2@C_{2n}(CH_2Ph)$ derivatives (Supplementary Figure 3). Despite the complexity, the spectrum clearly shows that $Y_2@C_{2n}(CH_2Ph)$ derivatives do have large ^{89}Y hfc constants of 65-80 G, which proves that the single-occupied Y-Y bonding MO is preserved in $Y_2@C_{2n}(CH_2Ph)$ derivatives. Note also that the sharp peak near 3490 G indicates that non-Y organic radical are also formed in the reaction. However, it is not practical and not essential for this work to give a detailed interpretation of the whole complex spectrum; subsequent HPLC separation of this mixture gave the pure **Y_2-I** compound, which has simple triplet spectrum and does not contain any other radical impurities (see Figure 3 in the manuscript).

Supplementary Note 2. Estimation of the relative yield of M_2-I

Dy_2-I

Assuming the extinctions coefficients of all the fullerenes at 320 nm are the same, then the amount of the fullerenes is linearly related to the HPLC peak area.

$$\frac{\text{HPLC peak area of FrA}}{\text{HPLC peak area of all the extracted fullerenes}} = \frac{783.02}{4653.89} \approx 16.82\%$$

$$\frac{\text{HPLC peak area of FrA-4}}{\text{HPLC peak area of FrA}} = \frac{1079.03}{3742.36} \approx 28.83\%$$

$$\frac{\text{HPLC peak area of FrA-4-2}}{\text{HPLC peak area of FrA-4}} = \frac{1197.39}{2640.85} \approx 45.34\%$$

$$\frac{\text{HPLC peak area of FrA-4-2-3}}{\text{HPLC peak area of FrA-4-2}} = \frac{262.16}{323.01} \approx 81.16\%$$

So, the relative yield of Dy_2-I to all the extracted fullerenes is

$$0.1682 \times 0.2883 \times 0.4534 \times 0.8116 \approx 2\%$$

With about 180 g graphite and 60 g Dy_2O_3, we obtained about 2.5 mg Dy_2-I.

For Y_2-I, similar estimation is giving following:

$$\frac{\text{HPLC peak area of FrA}}{\text{HPLC peak area of all the extracted fullerenes}} = \frac{761.72}{2538.01} \approx 30.01\%$$

$$\frac{\text{HPLC peak area of FrA-4}}{\text{HPLC peak area of FrA}} = \frac{1934.27}{4826.12} \approx 40.08\%$$

$$\frac{\text{HPLC peak area of FrA-4-2}}{\text{HPLC peak area of FrA-4}} = \frac{1260.78}{2866.42} \approx 43.98\%$$

The relative yield of Y_2-I to all the extracted fullerenes is

$$0.3001 \times 0.4008 \times 0.4398 \approx 5\%$$

Supplementary Note 3. X-ray crystallographic analysis of Dy_2-I

For the single crystal growth, about 0.1 mg Dy_2-I was dissolved in toluene, hexane was layered over the toluene solution, several single crystals was prepared successfully, the dimension of the picked single crystal for the XRD is 0.03 x 0.03 x 0.01 mm^3. Crystal data and data collection parameters are summarized in Supplementary Table 1.

The Dy_2-I density in the single crystal could be calculated as following:

$$Crystal\ Density = \frac{weight\ of\ two\ Dy_2I\ molecule}{Volume\ of\ the\ cell} = \frac{2 \times 1377 g/mol}{2264 Å^3}$$

$$= \frac{2 \times 1377 \times 1000 mg/mol}{6.02 \times 10^{23} mol^{-1} \times 2264 \times 10^{-21} mm^3} \approx 2.02 mg/mm^3$$

$$mass\ of\ Dy_2I\ in\ the\ picked\ single\ crystal \approx \frac{2.02mg}{mm^3} \times 0.03 \times 0.03 \times 0.01 mm^3 \approx 18ng$$

There is some disorder with the Dy atoms, two positions of one Dy atom were refined with site occupancies of 0.6983(17) and 0.3016(17) for Dy1A and Dy1B, respectively; three positions for another Dy atom were refined with site occupancies of 0.657(2), 0.2910(18) and 0.052(2) for Dy2A, Dy2B and Dy2C, respectively (see Supplementary Figure 11). Two pairs of Dy positions could be used to evaluate the interaction between the two encapsulated Dy atoms based on the site occupancies. The distances for Dy1A-Dy2A and Dy1B-Dy2B are 3.8965(14) and 3.898(3) Å, respectively. The encapsulated Dy atoms are coordinated to hexagons (highlighted green in Supplementary Figure 11) of the fullerene cage in a quasi-η^6 fashion with Dy–C distances in the coordinated hexagon being 2.308(8)-2.586(9) Å.

Supplementary Figures 12 and 13 show the packing of the **Dy_2-I** molecules in the single crystal, indicating that the Dy ions in the crystal are well aligned. Fullerene molecules form quasi-hexagonal layers with an AAA staking sequence. Toluene molecules and benzyl groups occupy the voids between the layers. The closest distances between centroids of fullerene fragments within the hexagonal layer are 10.89/10.97/11.11 Å. The distance between the layers is 10.98 Å, and the shortest distance between centroids of fullerene fragments from different layers is 11.04 Å. Some of the neighboring fullerenes within the layer face each other by the hexagons, coordinated by Dy1A/Dy2A atoms with larger occupancies (~0.7). The distance between centroids of these hexagons is 3.55 Å. The distances between Dy atoms of such neighboring molecules are 7.31/7.33 Å (compare to 3.90 Å for the intramolecular Dy–Dy distance).

Supplementary Note 4. Spectroscopic characterization of Dy_2-I and Y_2-I

UV-Vis-NIR absorption spectra of EMFs are dominated by π–π* excitation in the fullerene cage. Therefore, the spectra are very sensitive to the fullerene structure, but are almost insensitive to the encapsulated metals. Close similarity of the spectra of EMFs with different metal atoms may serve as a proof of the structural similarity. Absorption spectra of **Y_2-I** and **Dy_2-I** are very similar (Supplementary Figure 16), proving that the two compounds have the same fullerene cage structure. Absorption features are extended to about 1100 nm, which indicates that the **M_2-I** molecules have a relatively large optical gap exceeding 1.1 eV despite their open-shell electronic character. The spectra are also similar to that of La_2-I from ref.[1]

Similar to the absorption spectra, vibrational spectra of EMFs are also dominated by the vibrations of the fullerene cage. Therefore, close similarity of the vibrational spectra of two EMFs with different metals shows that the two EMFs have the same fullerene cage structure. Due to large masses of endohedral metal atoms, metal-based vibrations occur at low frequencies (below 200 cm^{-1}) and can be detected in Raman spectra. Here the difference in the masses of metal atoms is seen as the shift of the characteristic vibrational bands.

Supplementary Figures 17 and 18 show that the IR and Raman spectra of **Y_2-I** and **Dy_2-I** are virtually identical, which additionally confirms that isolated **Y_2-I** and **Dy_2-I** have the same fullerene cage structure. The only noticeable differences are the Raman bands marked with red arrows, which have noticeably different frequencies in **Y_2-I** (181 cm^{-1}) and **Dy_2-I** (148 cm^{-1}). They are assigned to the meal-cage stretching vibration, and significant metal-dependent shift is due to the larger mass of Dy (162.5 amu) than that of Y (88.9 amu).

Supplementary Note 5. Magnetic moment of Dy$_2$-I

The sample for the measurements of magnetic properties was drop-casted from CS_2 solution into propylene capsule and dried under vacuum overnight. The mass of the sample was determined by the change of the mass of the capsule before and after drop-casting/drying, each mass measurement was performed three times:

The weight of the empty capsule: 0.19014 g, 0.19014 g, 0.19013 g;

The weight of the capsule with **Dy$_2$-I**: 0.19101 g, 0.19101 g, 0.19102 g.

The mass of the sample used for magnetic measurements is thus determined to be **0.88 mg** with weighing uncertainty of 0.01 mg.

Saturated magnetization of the sample at 2 K in the field 7 T is 0.0372 emu, which gives the magnetic moment of 10.5 μ_B per molecule. Taking into account that the measurement are performed for a disordered powder sample, the value should be doubled, giving the moment of **20.9 μ_B** per $Dy_2@C_{80}(CH_2Ph)$ molecule. Uncertainty of the mass of 0.01 mg gives uncertainty of the moment of 0.2-0.3 μ_B. 20.9 μ_B is very close to 21.0 μ_B, theoretical value for the [Dy^{3+}–*e*–Dy^{3+}] system with collinear ferromagnetically coupled moments of two Dy ions and one unpaired electron. In case of antiferromagnetic coupling between magnetic moment of Dy and unpaired spin, the total moment of the molecule would be 19.0 μ_B.

Supplementary Note 6. Determination of relaxation times from decay curves

Long magnetization relaxation times (> 10 sec) were determined from the measurement of magnetization decay using dc-SQUID. The sample was first magnetized to the saturation at 5 Tesla, then the field was swept as fast as possible to zero or 0.4T, and then the decay of magnetization was followed over several hours. Decay curves could not be described by single exponent and were then fitted using stretched exponential function:

$$Y_{stretched} = A_0 e^{-\left(\frac{t}{t_0}\right)^b} + y_0,$$

where t_0 is the relaxation time and y_0 is an equilibrium magnetization at the given field and temperature. For the in-field measurements with very long relaxation times, reliable determination of y_0 parameter was crucial and could not be accomplished from the decay curve alone. In such cases, the second curve was measured by cooling the sample first in zero-field, then applying the desired finite field (0.4 T), and then following increase of magnetization with time (Supplementary Figure 23). Both decay and growth curves should end-up in the same magnetization value equal y_0, and hence the curves were fitted together. Relaxation times determined in dc measurements are listed in Supplementary Tables 2 and 3.

Supplementary Note 7. Contribution of different relaxation mechanisms to the relaxation of magnetization

Experimentally determined relaxation times were fitted using two equations, one for zero-field data (Equation (1)), another one for the measurements in the field of 0.4 T (Equation (2)). The equations are different only in the absence of the QTM term in Equation (2), which describes the relaxation in a finite field. Parameters of both equations were kept identical during the fit. Resulting fitting curves as well as contributions of individual relaxation processes are shown in Supplementary Figure 27.

We also attempted to describe the whole set of experimental points using only one Orbach process:

Zero-field: $$\tau_M^{-1} = \tau_{QTM}^{-1} + CT^n + \tau_0^{-1}\exp(-U^{eff}/T)$$ (Supplementary Equation 1)

In-field: $$\tau_M^{-1} = CT^n + \tau_0^{-1}\exp(-U^{eff}/T)$$ (Supplementary Equation 2)

The fit of experimental points obtained with Supplementary Equations (1) and (2) is substantially worse than with the use of Equation (1) and (2) in the main text, especially between 10 and 25 K (Supplementary Figure 28). Raman relaxation (C = 3.84·10^{-10} s^{-1}K^{-n}, n = 6.7) now dominates all low-temperature data for the in-field relaxation and is switched to the Orbach regime (U^{eff} =656 K, τ_{02}=8.9·10^{-13} s) above 23 K. Thus, we conclude that the terms included in Supplementary Equations (1) and (2) are not sufficient for the description of the whole set of data.

Supplementary Note 8. Ab initio and point-charge calculations of crystal field parameters

Full ab initio treatment of **Dy_2-I** is not possible at this moment, so we performed calculations for single Dy centers and replaced another Dy ions by Y. Besides, the unpaired valence electron was "quenched" by adding one extra electron to the system. Ab initio energies and wave functions of crystal-filed (CF) multiplets for the [**DyY-I**]$^{-}$ molecule (Supplementary Table 6) have been calculated using the quantum chemistry package MOLCAS 8.0. Single point complete active space self-consistent field with spin-orbit interactions calculations (CASSCF/SO-RASSI level of theory) were done to derive ab initio parameters. In all systems, the Dy(III) has $^6H_{15/2}$ ground state multiplet, which results in eight low-lying Kramers doublets. The active space of the CASSCF calculations includes nine active electrons and the seven active orbitals (e.g. CAS (9,7)). All 21 sextet states and only 108 quartets and 100 doublets were included in the state-averaged CASSCF procedure and were further mixed by spin-orbit coupling in the RASSI procedure. Mixed atomic natural extended relativistic basis set (ANO-RCC) was employed with the minimal basis option for C and H atoms, and VDZ-quality for Y and Dy metals in the cluster. The single ion magnetic properties and CF-parameters (Supplementary Table 7) were calculated with use of SINGLE_ANISO module. The energies and compositions of single-ion CF states of Dy1 and Dy2 are visualized in Supplementary Figure 29.

Model computations were performed with simplified system, in which the carbon cage was omitted, and only metal ions with additional point charges were included. In particular, we computed the [Dy-Y] "dimers" in their 6+ and 4+ charge states, as well as the [Dy-H-Y]$^{+5}$ "molecule". In [Dy–Y]$^{+6}$, the metal-metal bond is absent – hence this ion models the **[DyY-I]$^{+}$** system with the electron removed from the M-M bond. In [Dy–Y]$^{+4}$, there is a two-electron covalent bond between the metals, and hence this ion models the [**DyY-I**]$^{-}$ molecule. The [Dy-H-Y]$^{+5}$ is similar to the [Dy–Y]$^{+4}$, but has a proton added at the mid-point between Dy and Y; this system essentially simulates the charge distribution in the system with the single-electron bond between the metal, but lacks the complication induced by the presence of the unpaired spin. Computations for these model systems were performed either *ab initio* at the CASSCF level as described above or with the point-charge model using the McPhase code (Supplementary Tables 8, 9). Crystal field parameters from either *ab initio* or point-charge model calculations (Supplementary Table 10) were then transferred to the PHI code for the further analysis.

In a modeling with the PHI code, we have been using pseudospin model with total spin S=15/2, which produces eight Kramers doublets for a single center in presence of a CF field. The effective g-factors for the coupled spin multiplet were set 1.33 (assuming the $4f^9$-configuration). Transition probabilities were also computed by using the PHI code.

Supplementary Note 9. Crystal field Hamiltonian and Crystal-field potential

The complete crystal field part of Hamiltonian for each atomic center is defined as:

$$\widehat{\mathrm{H}}_{\mathrm{CF}} = \sum_{k=2,4,6} \sum_{q=-k}^{k} B_k^q \theta_k \hat{O}_k^q$$

where B_k^q are CF-parameters in Steven's notation and $\theta_k, \hat{O}_k^q$ the operator equivalent factors and operator equivalents respectively. In case of a simple linear system [Dy^{3+}–q_m–(+3)], the CF-Hamiltonian is dominated by the single term with k = 2 and q = 0, $\widehat{\mathrm{H}}_{\mathrm{CF}} = B_0^2 C_0^2$, where $B_0^2 = A_0^2 < r^2 >$ and $C_0^2 = \frac{1}{2}(3cos^2\vartheta - 1)$.

As the radial integral $< r^2 >$ is positive and for linear alignment ($\vartheta = \pi$) $C_0^2 = 1$, the sign of the $\widehat{\mathrm{H}}_{\mathrm{CF}}$ is determined by geometrical coordination factor $A_0^2 = -e\frac{q}{R^3}C_0^{-2} = -e\frac{q}{R^3}\frac{1}{2}\sqrt{\frac{3}{\pi}}cos\,\vartheta = \frac{1}{2}\sqrt{\frac{3}{\pi}}\frac{eq}{R^3}$. For two point charges with Q and q_m, the total CF-potential proportional to: $\frac{eq_m}{R_{q_m}^3} + \frac{eQ}{R_Q^3}$.

Visualization of the CF potential in Figure 5d and Supplementary Figure 30 shows that even rather small negative charge placed at the midpoint between Dy^{3+} ion and a 3+ point charge outweighs the effect of the positive charge. Supplementary Figure 30 shows the variation of the B_0^2 parameter and the change of its sign with the variation of the negative charge. As B_0^2 changes the sign with the increase of the negative charge, the anisotropy type of the Dy ion changes from easy-plane to easy-axis.

Supplementary Note 10. Exchange coupling in the M_2-I

The interactions between spin centers in the [M^{3+}–*e*–M^{3+}] system can be described with the following effective Hamiltonian:

$$\hat{H}_{ex-dip} = -2j_{12}\hat{J}_{M1}\cdot\hat{J}_{M2} - 2j_{1,e}\hat{J}_{M1}\cdot\hat{S}_e - 2j_{2,e}\hat{J}_{M2}\cdot\hat{S}_e \quad \text{(Supplementary Equation 3)}$$

Here we suggest that similar to the Lines model the interactions between the centers can be described by a set of isotropic exchange parameters where j_{12} (interactions between two metal centers) and $j_{1,e}$/$j_{2,e}$ (interactions between metal centers M1/M2 with the unpaired electro spin). The j_{12} parameter can be further subdivided into exchange and dipolar contributions, j_{12}^{ex} and j_{12}^{dip}. We do not take into account here that the *j* values may vary with the increase of J_z moments of lanthanides or that CF and exchange state may mix substantially by changing the interaction parameters. Development of the more rigorous interaction Hamiltonian might be needed to describe these effects, such the model proposed by Chibotaru et al. recently,[2] but it exceeds the scope of this paper. We also note that the use of more refined model for lanthanides coupled via strong exchange in the Ref. 2 led to significant changes in the transition probabilities by admixing exchange and CF states, but the energy spectrum, at least in its low-energy part, was not dramatically altered.

Theoretical prediction of the *j* constants in Supplementary Equation (3) for lanthanides is hardly possible at this moment, except for the [Gd^{3+}–*e*–Gd^{3+}] system, which can be modelled more reliably. To estimate a possible strength of the exchange coupling in **Gd_2-I**, we performed broken-symmetry DFT calculations at the PBE0/TZVP level using Orca package. The high-spin state with all spins aligned is found to be the ground state (see Supplementary Figure 31 for the spin density distributions). Flipping the spin on one of the Gd centers gives two broken-symmetry states with the energies of 1212 and 1231 cm^{-1} (the metals are slightly different because of the non-symmetric position of the benzyl group at the cage), whereas the energy of the state obtained by flipping the spin on the Gd–Gd bond is 2555 cm^{-1}. Mapping these values on the Hamilton in Supplementary Equation (3) gives exchange couplings between Gd and the single electron spin of 181 and 184 cm^{-1} (250 and 254 K), and direct Gd–Gd coupling of −1.2 cm^{-1} (Supplementary Figure 31e). These values are quite similar to those predicted for $Gd_2@C_{79}N$ and represent the highest exchange coupling values known for lanthanide compounds. Although these values cannot be directly transferred to other lanthanides, at least the order of the value may be expected to be similar.

For anisotropic lanthanides with collinear alignment of their magnetic moments, the j_{12} constant also has j_{12}^{dip} term, which can be estimated straightforwardly using the following equation:

$$j_{12}^{dip} = -\frac{E^{dip}(\uparrow,\uparrow) - E^{dip}(\uparrow,\downarrow)}{2J_{z,1}J_{z,2}}$$

where E^{dip} is the energy of the dipole–dipole interaction between magnetic moments $\boldsymbol{\mu_1}$ and $\boldsymbol{\mu_2}$ at a distance R_{12} and it is defined as:

$$E^{dip}(\vec{\mu}_1,\vec{\mu}_2) = -\frac{\mu_0}{4\pi R_{12}^3}\left(3(\vec{n}_r\cdot\vec{\mu}_1)(\vec{n}_r\cdot\vec{\mu}_2) - (\vec{\mu}_1\cdot\vec{\mu}_2)\right)$$

where $\vec{n}_r$ is the normal of the radius vector connecting two magnetic moments $\vec{\mu}_1$ and $\vec{\mu}_2$. According to *ab initio* and simple point charge calculations, the ground state for each Dy^{3+} center in **Dy_2-I** is an easy-axis state with μ_i = 10 μ_B (J_z = ±15/2, g = 1.33). The dipolar energy difference between parallel and antiparallel alignment of these moments at the distance of 3.96 Å is 4.0 K (2.77 cm^{-1}), which gives j_{12}^{dip} = 0.012 cm^{-1}.

Computations of exchange interactions in **Gd_2-I** and estimation of the dipolar term in **Dy_2-I** show that that the strength of the direct coupling between two metal centers, j_{12}, is very small compared to the metal-electron interactions ($j_{i,e}$) and in the first approximation can be neglected.

Supplementary Note 11. Magnetic susceptibility of Dy_2-I and spin Hamiltonian parameters

In this section we will simulate $\chi_m T$ curves with different parameters of the crystal field and exchange interactions and compare results of simulations to the experimentally measured $(M/B)_m T$ functions. The temperature and field dependence of the $(M/B)_m T$ function is shown in Supplementary Figure 32. At low magnetic fields, the function is similar to $\chi_m T$, but in high fields the difference between the derivative $\partial M/\partial B$ and the ratio M/B is significant. This can be clearly seen in Supplementary Figure 33, which compares $\chi_m T$ and $(M/B)_m T$ functions computed for different magnetic fields. The two functions deviate significantly when the external field exceeds 1 Tesla.

In all magnetic fields studied, $(M/B)_m T$ shows a sharp increase to ca 55 $cm^3mol^{-1}K$ with the increase of the temperature. At higher temperatures, the function decreases slowly reaching ca 43 $cm^3mol^{-1}K$ at 300 K. But for the low external fields, the low-temperature part of the curve is disturbed by slow relaxation of magnetization (the kink in the 0.2 T curve corresponds to the blocking temperature). For the fields exceeding 1 T the whole curve is measurable. For this reason, and because in the field of 1 T the $(M/B)_m T$ function is still close to the $\chi_m T$ function, in analysis of the Hamiltonian parameters below we will use the $(M/B)_m T$ measured in the field of 1 T and compare it to the computed $\chi_m T$ curves.

In the simulations described below, the **Dy_2-I** system was modelled using the following effective spin Hamiltonian (Equation (4) of the main text):

$$\hat{H}_{tot} = \hat{H}_{CF(Dy1)} + \hat{H}_{CF(Dy2)} - 2j_{Dy,e}(\hat{J}_{Dy1}\cdot\hat{S}_e + \hat{J}_{Dy2}\cdot\hat{S}_e)$$

To determine the plausible values of the $j_{Dy,e}$ constant, the $\chi_m T$ curves were simulated with the *ab initio* calculated CF parameters (see Supplementary Note 8) and with different $j_{Dy,e}$ constants from 5 to 40 cm^{-1}. Supplementary Figure 34 shows that small $j_{Dy,e}$ values lead to the sharp peak at low temperatures in the predicted $\chi_m T$ curves. With the increase of the $j_{Dy,e}$ constant, the peak is becoming broader and is shifting towards higher temperatures. Reasonable agreement with experimental $(M/B)_m T$ measured in the field of 1 T is obtained for the $j_{Dy,e}$ constant of 30–35 cm^{-1}.

To evaluate the effect of the single-ion anisotropy, we fixed $j_{Dy,e}$ constant to 30 cm^{-1} and created different sets of CF parameters using the point-charge model described in section Supplementary Note 8 and in Figure 5d of the main text. In particular, Dy ions were placed in the crystal field created by a positive charge of +3 at the distance of 3.96 Å and with the negative charge at the midpoint between Dy ion and the positive charge. Variation of this negative point charge from −0.4 *e* to −2.0 *e* gave a set of easy-axis CF parameters with the increasing CF splitting. Supplementary Figure 35 compares the experimental curve to the results of simulations. The small CF splitting results in the peak of the $\chi_m T$ function at low temperature, which is shifting to higher temperature with the increase of the CF splitting. Good agreement between experiment and theory is obtained when the splitting between the ground state and the first excited CF state exceeds 200 cm^{-1}. The lower limit of 200 cm^{-1} agrees well with the results of CASSCF calculations (which give very similar curve to the point charge model with the negative charge of −0.8 *e*).

To summarize, these simulations show that the shape of the experimental curve requires the exchange constant higher than 30 cm^{-1} and the crystal field with the splitting of the first two CF states exceeding 200 cm^{-1}. If any of these parameters are smaller, the $\chi_m T$ function has a well-defined peak at low temperature, which contradicts the experimental data due the presence of the low-energy excited state with lower magnetic moment, whose thermal population decreases $\chi_m T$.

Supplementary Note 12. Magnetization curves at different temperatures

Magnetic moment of 21 μ_B per molecule determined from the saturation of magnetization at low temperatures indicates that magnetic moment of Dy ions and of the unpaired electron are coupled ferromagnetically. In case of antiferromagnetic (AFM) coupling between Dy and unpaired electron spin, the total moment per molecule would be 19 μ_B. An alternative proof of the FM coupling in the [Dy^{3+}–*e*–Dy^{3+}] system can be obtained from the shape of the magnetization curves. To exclude the possible errors in the mass determination, comparison was done for the curves normalized to the magnetization value measured at 1.8 K in the field of 7 T.

Supplementary Figure 36 shows magnetization curves of **Dy_2-I** measured at 1.8, 20, 25, 30, 50, 40, 70, and 100 K (note the broad hysteresis at 1.8 K, which still remains open at 20 K).

The curves were computed using spin Hamiltonian in Equation (4) with the $j_{Dy,e}$ value of +32 cm^{-1} (FM coupling) and −32 cm^{-1} (AFM coupling); $j_{Dy,Dy}$ was assumed to be 0 in both cases. Note that in the spectrum of the spin Hamiltonian, the ground state is separated from the first excited state by more than 240 cm^{-1}, which ensures that magnetization curves measured at temperatures up to 100 K are dominated by the ground state properties. The difference between 19 and 21 μ_B is not well seen at low temperatures (simulated curves are almost identical at 1.8 K), but the curves diverge more pronouncedly at 35–100 K. Comparison to the experimental data shows that the FM coupling describes the system considerably better than the AFM coupling, which serves as an independent proof of the magnetic moment of 21 μ_B.

Supplementary Note 13. Spectrum of the spin Hamiltonian and properties of the eigenstates

Computations of the CF parameters and estimation of the coupling parameters allow us to compute and analyze the spectrum of the spin Hamiltonian (Equation (4) in the main text):

$$\hat{H}_{tot} = \hat{H}_{CF(Dy1)} + \hat{H}_{CF(Dy2)} - 2 j_{Dy,e} (\hat{J}_{Dy1} \cdot \hat{S}_e + \hat{J}_{Dy2} \cdot \hat{S}_e)$$

Supplementary Figure 37 shows the whole spectrum, whereas Figure 5b in the manuscript shows enhancement of the low-energy range (0–1000 cm^{-1}). In calculations we used $j_{Dy,e}$=32 cm^{-1}, and *ab initio* computed CF parameters.

The whole spectrum is rather complex and spans the energy range of 0–3000 cm^{-1}. Taking into account the simple form of the effective spin Hamiltonian, we think that the high-energy range can hardly be described reliably and requires more rigorous treatment of the exchange interactions and mixing of CF and exchange. Yet, for the low-energy part of the spectrum, describing the states with predominantly a collinear moments, the reasonable description of the system is still expected. Good agreement between experimental and calculated $(M/B)_m T$ curves ensures that at least in the low-energy range, the effective spin Hamiltonian provides reasonable description of the system. Description of the eigenvectors of the Hamiltonian in the basis of the J_z states of individual centers is given in Supplementary Table 11. Supplementary Table 12 lists pseudospin g-tensors for several lowest-energy states.

The strong exchange interactions in the [Dy^{3+}–*e*– Dy^{3+}] system and moderate CF spitting leads to quite an unusual situation, as the lowest-energy CF excited state have lower energy than the exchange state. Two first excited states correspond mainly to the excitation of J_z state of one of Dy centers to J_z=±13/2. With two non-equivalent Dy centers, we have two such states in **Dy_2-I**, at 241 and 302 cm^{-1}. Two further CF-excited state are found at 412 and 436 cm^{-1}, whereas at 428 and 434 cm^{-1} two exchange-excited states are found (again, the two states have slightly different energies because Dy ions are not completely equivalent). In these states, the leading term corresponds to the configuration with both Dy centers in their J_z=±15/2 state, but with the antiparallel alignment (i.e. the spin of one of the Dy centers is flipped).

Analysis of the transition probabilities show that direct transition between CF-excited states is unlikely. However, there is a certain probability between the second CF-excited state at 436 cm^{-1} and the exchange-excited state. Transitions between two exchange excited states of opposite spin have rather high probabilities. Furthermore, the barrier of the Orbach relaxation process, 613 K, fits well into the expected energies of the exchange excited states. Based on these facts, we propose that the high-temperature Orbach process corresponds to the exchange excited state with the energy of 613 K, which gives the $j_{Dy,e}$ value of 32 cm^{-1}.

Supplementary References

1. Bao L, Chen M, Pan C, Yamaguchi T, Kato T, Olmstead MM*, et al.* Crystallographic Evidence for Direct Metal–Metal Bonding in a Stable Open-Shell $La_2@I_h$-C_{80} Derivative. *Angew Chem-Int Edit Engl* 2016, **55**(13)**:** 4242-4246.

2. Vieru V, Iwahara N, Ungur L, Chibotaru LF. Giant exchange interaction in mixed lanthanides. *Sci Rep* 2016, **6:** 24046.
